# Megaphages infect *Prevotella* and variants are widespread in gut microbiomes

**DOI:** 10.1038/s41564-018-0338-9

**Published:** 2019-01-28

**Authors:** Audra E. Devoto, Joanne M. Santini, Matthew R. Olm, Karthik Anantharaman, Patrick Munk, Jenny Tung, Elizabeth A. Archie, Peter J. Turnbaugh, Kimberley D. Seed, Ran Blekhman, Frank M. Aarestrup, Brian C. Thomas, Jillian F. Banfield

**Affiliations:** 1 Department of Earth and Planetary Science, University of California, Berkeley, Berkeley, CA USA; 2 Institute of Structural & Molecular Biology, Division of Biosciences, University College London, London, UK; 3 Department of Plant and Microbial Biology, University of California, Berkeley, Berkeley, CA USA; 4 National Food Institute, Technical University of Denmark, Lyngby, Kongens, Denmark; 5 Department of Evolutionary Anthropology, Duke University, Durham, NC USA; 6 Department of Biological Sciences, University of Notre Dame, Notre Dame, IN USA; 7 Department of Microbiology and Immunology, University of California, San Francisco, San Francisco, CA USA; 8 Chan Zuckerberg Biohub, San Francisco, CA USA; 9 Department of Genetics, Cell Biology, and Development, University of Minnesota, Minneapolis, MN USA; 10 The University of Melbourne, Melbourne, Victoria Australia; 11 Innovative Genomics Institute, University of California, Berkeley, Berkeley, CA USA; 12 Present Address: Department of Bacteriology, University of Wisconsin–Madison, Madison, WI USA

**Keywords:** Bacteriophages, Microbial communities

## Abstract

Bacteriophages (phages) dramatically shape microbial community composition, redistribute nutrients via host lysis and drive evolution through horizontal gene transfer. Despite their importance, much remains to be learned about phages in the human microbiome. We investigated the gut microbiomes of humans from Bangladesh and Tanzania, two African baboon social groups and Danish pigs; many of these microbiomes contain phages belonging to a clade with genomes >540 kilobases in length, the largest yet reported in the human microbiome and close to the maximum size ever reported for phages. We refer to these as Lak phages. CRISPR spacer targeting indicates that Lak phages infect bacteria of the genus *Prevotella*. We manually curated to completion 15 distinct Lak phage genomes recovered from metagenomes. The genomes display several interesting features, including use of an alternative genetic code, large intergenic regions that are highly expressed and up to 35 putative transfer RNAs, some of which contain enigmatic introns. Different individuals have distinct phage genotypes, and shifts in variant frequencies over consecutive sampling days reflect changes in the relative abundance of phage subpopulations. Recent homologous recombination has resulted in extensive genome admixture of nine baboon Lak phage populations. We infer that Lak phages are widespread in gut communities that contain the *Prevotella* species, and conclude that megaphages, with fascinating and underexplored biology, may be common but largely overlooked components of human and animal gut microbiomes.

## Abstract (web summary)

Here the authors discover megaphages, or Lak phages, with genomes >540 kilobases in length in the gut microbiomes of a range of hosts, identify *Prevotella* as the bacterial host and suggest that these phages are common members of the mammalian gut microbiome.

## Main

Human and animal microbiomes are of enormous interest, given that microbial activity impacts nutrition, physiological development and disease^1^. The human gut microbiome has been intensively studied, mostly using gene fingerprinting methods to resolve body site specificity and microbiome compositional changes with age, health conditions and diet^2–4^. Less commonly applied is genome-resolved metagenomics, which involves simultaneous recovery of draft, and sometimes complete, genomes from metagenomes. Such studies have provided access to bacteriophage (phage), virus and plasmid sequences that are not accessible via fingerprinting methods^5–7^.

Phages are increasingly recognized as ubiquitous components of microbiomes. They can dramatically shape ecosystem structure via strain-specific predation, mediate horizontal gene transfer and redistribute nutrients by lysing host cells^8^. We investigated microbial communities in the gastrointestinal tracts of ten arsenic-impacted men from Laksam Upazila, Bangladesh to identify gut microbiome-associated phages, link them to bacterial hosts and evaluate their distribution. We discovered phages with genomes that are exceptionally large, >540 kilobase pairs (kb) in length (referred to as ‘megaphages’ in this study). As of June 2016, only 93 phages with genomes >200 kb (‘jumbo phages’) were isolated, and none have a genome >500 kb^9^. The average length of complete phage genomes is 53,644 ± 45,677 bp, consistent with the average length of isolated double-stranded DNA viruses (44,296 ± 83,777 bp)^10^. We refer to the megaphages discovered in the Laksam Upazila cohort as Lak phages and determined that they replicate in *Prevotella* species, bacteria that tend to be enriched in the gut microbiomes of individuals who consume non-Western diets^11^. To determine whether these phages are common in other human and animal microbiomes, we investigated several DNA read data sets from samples containing abundant *Prevotella*. Overall, our results indicate that Lak phages are common and probably important components of the gut microbiomes of humans and animals.

## Results

### Megaphages identified in the gut microbiomes of Bangladeshi adults

We sequenced DNA from the faecal samples of ten adults living in Eruani village, Laksam Upazila, Bangladesh (Supplementary Table 1). Taxonomic classification and relative abundance information reveal that the communities are mostly dominated by *Prevotella* species (Supplementary Fig. 1). From individuals 20 and 22 we identified large genome fragments that were identified as phages (Supplementary Information) and selected for manual assembly curation (Methods). Two bioinformatically verified, circularized phage genomes, A1 and A2, were >541 kb in length, close to the maximum size ever reported for a phage^10^. There was no evidence for integration of these sequences into bacterial genomes (Supplementary Information). Given their extraordinary size and to distinguish them from jumbo phages (>200 kb genomes^9^), we refer to these as ‘megaphages’. The A1 and A2 genomes are largely syntenic and share 91.3% average nucleotide identity (ANI). They encode ~35 putative transfer RNA (tRNA) genes (Table 1), many of which are concentrated in specific genomic regions. Four near-identical A1 phage genomes were independently curated to completion from samples taken on consecutive days from individual 22 (Table 1). The identified sequence variation occurred as population heterogeneity in all samples. Variant frequency analysis confirmed that the polymorphic sites are not linked; shifting relative abundances of subpopulations suggests ongoing replication over the four days (Supplementary Fig. 2).

**Table 1:** Complete megaphage genomes (see also Supplementary Table 1)

Phage	Sample of origin	Guanine-cytosine, %	Length (bp)	No. tRNAs	No. tRNA introns	No. predicted open reading frames (code 15)
A1-i	As cohort 22, no. 2	25.9	541,643	33	3	581
A1-ii	As cohort 22, no. 3	25.9	541,664	33	3	581
A1-iii	As cohort 22, no. 4	25.9	541,664	33	3	584
A1-iv	As cohort 22, no. 5	25.9	541,664	33	3	581
A2	As cohort 20, no. 3	26.0	541,299	34	4	581
C1	Cholera CH_A02_001D1	25.8	540,217	32	2^a^	591
B1	Baboon F22 (V)	26.0	547,991	30	1	591
B2	Baboon F3 (V)	26.0	549,839	31	1	594
B3	Baboon M09 (V)	26.0	546,746	30	1	590
B4	Baboon F30 (V)	26.0	550,552	31	1	594
B5	Baboon F18 (V)	26.7	543,529	31	1	583
B6	Baboon F16 (V)	25.8	546,689	30	1	588
B7	Baboon F11 (M)	26.0	550,702	31	1	599
B8	Baboon F4 (V)	26.0	551,627	31	1	600
B9	Baboon F01 (V)	26.0	550,053	30	1	593

^a^Variants within incomplete genomes have tRNA introns not found in the C1 genome. Baboons are from two social groups, V (Viola’s) and M (Mica’s) (see Tungi et al.^16^).

### *Prevotella* species are predicted as the hosts for megaphages based on CRISPR targeting

**Fig. 1:**
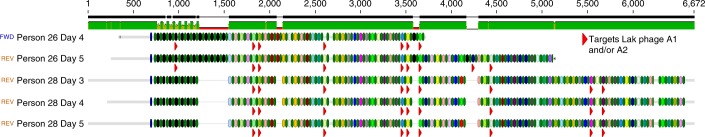
Alignment of the CRISPR arrays on four *Prevotella* scaffolds containing repeat GGTTTAATCGTACCTTTATGGAATTGAAAT. The green rods indicate repeats, the coloured rods indicate spacers. The same colour indicates the same spacer sequence, except for black rods, which indicate spacers different between individuals 26 and 28 (probably added to the diversifying locus ends). The red arrows indicate spacers targeting megaphages (also see Supplementary Fig. 3).

We used CRISPR targeting^12^ to identify *Prevotella* as the megaphage host (Fig. 1 and Supplementary Fig. 3, Supplementary Information). Given that many of the individuals have gut microbiomes dominated by *Prevotella*, we tested for megaphages in all the Laksam Upazila microbiome samples and found evidence for them in samples from individuals 21 and 23 (Supplementary Information). We attempted to isolate the megaphages using faecal material and *Prevotella copri* DSM 18205^13^ but isolation was unsuccessful (Supplementary Information).

### Megaphages occur in other gut microbiomes

In a prior study, faecal samples were collected from a cohort of Bangladeshi cholera patients who were hospitalized in Dhaka, Bangladesh in 2016, but the reads were not assembled^4^. We conducted genome-resolved metagenomic analyses of these data sets. Many of the gut microbiomes were dominated by *Prevotella* and contained phages related to the A1 and A2 megaphages. One 540,217 kb genome, C1, was manually curated to completion. A data set from a second Bangladeshi cohort comprising six cholera-impacted adults was sampled from the same hospital in 2011. Of these, S75 had relatively abundant phages related to C1, and S71 and S72 had >100 reads map to the C1 genome (Supplementary Fig. 4).

Faecal samples from individuals from the Hadza tribe of Tanzania were sequenced in a prior study^14^. Three of the 27 Hadza individuals had megaphages in sufficiently high abundance for genome assembly (Supplementary Fig. 4). Our assemblies were highly fragmented, but sequences shared ~90% identity to phage A1 (Supplementary Table 1). Two samples from a previously sequenced cohort of Indian children^15^ also contained evidence of the megaphages (reads covered >50 kb of the A1 genome).

Previously published metagenomic shotgun sequencing data sets from the faecal samples of 48 members of two social groups of Kenyan yellow baboons (*Papio cynocephalus*; one metagenome per individual) were assembled and investigated to identify megaphage sequences^16^. Megaphages were detected in 43 of the 48 baboon gut microbiomes, and all samples contained multiple *Prevotella* strains or species (Supplementary Fig. 5). Sixteen high-quality genome bins were identified from 16 distinct samples, nine of which were curated to completion (B1–B9). All genomes were >543 kb in length, and one (B8) is the largest phage genome reported in this study (551,627 bp). All encode either 31 or 32 putative tRNAs (Table 1, Supplementary Table 2 and Supplementary Information).

We analysed sequence data from *Prevotella*-containing samples from Danish pigs (Supplementary Table 1). Despite genome fragmentation, we identified a total of 18.7 mega base pairs (Mb) of megaphage sequences with an alignment length of 15.9 Mb to the A1 genome (one bin comprises a 462 kb sequence). At least 2 kb of an aligned megaphage sequence was detected in 104 of the 105 metagenomes. The pig-derived sequences span the A1 genome (Supplementary Fig. 6c). Thus, megaphages related to those present in humans and baboons also colonize pigs. Reads from 4 of 27 cow rumen metagenomes^17^ mapped with low coverage across the entire A1 phage genome (Supplementary Table 1). However, analyses of 34 *Prevotella*-rich metagenomes from faecal samples from Tunapuco, a traditional agricultural community in the Andean highlands, did not detect megaphages^18^.

We identified a diversity of *Prevotella* strains via 16S ribosomal RNA (rRNA) gene phylogenetic analysis. However, we found no clear link between cohort type and *Prevotella* species, or *Prevotella* species and megaphages (Supplementary Fig. 7).

### Megaphage use an alternative genetic code

**Fig. 2:**
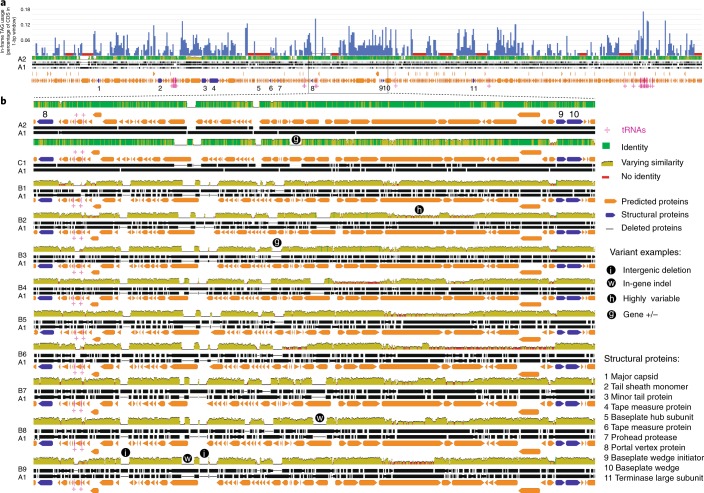
Genomic features and variation in Lak phage genomes. **a**, Frequency of use of the TAG repurposed stop codon overlying the alignment of the A1 and A2 genomes. The red bars indicate regions >5 kb without TAG codon use. Structural proteins (dark blue) are mostly encoded in regions with high TAG use. CDS, coding sequence. The boxed region is shown in detail in **b**. **b**, Alignments of each distinct Lak phage genome against the A1 genome. A subset of this region was used in the pig Lak phage genome fragment alignments (Supplementary Fig. 6).

A notable feature of the megaphage genomes was their low coding density (<70% for A1 and A2) when genes were predicted using the normal bacterial code (code 11). Fragmentation of many predicted proteins indicated that megaphages might be using an alternative genetic code. We determined that the canonical TAG stop codon is probably repurposed to encode glutamine, Q (code 15, Supplementary Fig. 8, see Methods), and confirmed this using the Fast and Accurate genetic Code Inference and Logo tool^19^. Code 15 was once previously reported for phages from metagenomes^20^. The TAG codon is not used in large parts of the A1 and A2 genomes, but it is used in some regions, including most that encode structural proteins (Fig. 2a and Supplementary Fig. 9). In samples from all days, we detected expression in regions encoding genes that do and do not use the TAG codon. Thus, if genes encoding structural proteins are expressed late, the phages in each sample are in a variety of stages of replication (Supplementary Fig. 9D).

Genomes with repurposed stop codons typically encode a suppressor tRNA. Multiple types of suppressor tRNAs were predicted (Supplementary Information and Supplementary Table 2), including one with a CTA anticodon that is necessary to repurpose the TAG stop codon. All complete megaphage genomes also encode release factor 2, which terminates translation by recognizing the TGA and TAA, but not TAG, stop codons. Thus, megaphages have the cellular machinery necessary to successfully translate genes with in-frame recoded TAG.

### Comparative megaphage genomics

Terminase proteins are important during capsid assembly. Based on phylogenetic analyses, megaphage terminases place generally within the Myoviridae. Since they are clearly a divergent clade and highly distinct in terms of their consistently very large genomes and use of alternative coding, we define them as the ‘Lak phages’, named after Laksam Upazila, Bangladesh from where they were first detected.

The A1, A2 and C1 genomes are syntenic, as are all baboon Lak (B-Lak) genomes, but six large rearrangements distinguish the B-Lak from the A-Lak and C1-Lak genomes (Supplementary Fig. 10). As expected based on their synteny, A1 and A2 are more similar to C1 than the B-Lak genomes (Supplementary Table 3, Supplementary Fig. 11). Alignment of ~70 kb region from each genome with the A1 genome (Fig. 2b) shows that insertions/deletions of sequence blocks within the central regions of genes (also see Supplementary Fig. 12), complete gene insertion/deletions, intergenic insertions/deletions and varying levels of nucleotide substitutions (sometimes varying greatly within a gene, Supplementary Fig. 12) distinguish the genomes.

The nine B-Lak genomes share ANI values between 88.5 and 99.9% with one another (Supplementary Table 3). For comparison, A1 and A2 share ~95% ANI (Supplementary Table 3). However, over an alignment with A1 (Fig. 2), B-Lak genomes share ~61–65% ANI and C1 share ~88% ANI. Notably, the majority of pig Lak genome fragments share >90% sequence identity with A1, genome-wide (Supplementary Fig. 6).

**Fig. 3:**
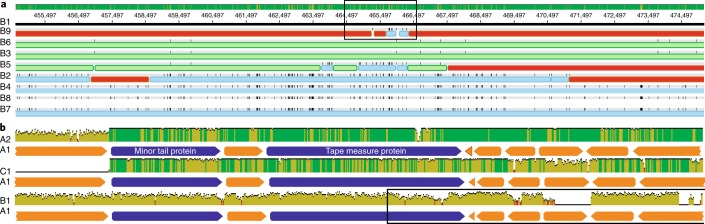
Comparison of B-Lak phage genomes reveals identical sequence blocks in a subset of the B-Lak phage. **a**, Sequence variation in a ~20 kb region of the aligned B-Lak phage genomes with B1 as the reference. The coloured bars underline blocks with a shared sequence. Note evidence of admixture of sequence blocks, indicative of extensive homologous recombination among phages sampled from individual baboons. For the full alignment of the nine complete B-Lak phage genomes, see Supplementary Fig. 13. The box indicates the B9 region examined in detail (Supplementary Fig. 14). **b**, Relatively conserved and divergent regions in A1, A2 and C1. The open box indicates the region shown in **a**.

Comparison of B-Lak genomes with one another revealed identical sequence blocks up to tens of kilobases in length in a subset of the B-Lak phage (Supplementary Fig. 13 and Fig. 3a). However, in adjacent regions, hundreds of single nucleotide polymorphisms (SNPs) distinguish these genotypes. These divergent sequences are often shared by a different subset of B-Lak genomes. The strong signal of sequence block admixture clearly indicates reassortment.

### Lak phage populations are near-clonal, but some contain sequences of different Lak phages

We analysed sequence variation in reads mapped to each B-Lak genome (Supplementary Table 4) and found that 94–96.4% of the reads map to the genome with zero SNPs, providing confidence that the reported genomes are not chimeras of population variants. However, 0.01–0.8% of reads and read pairs in each data set match the sequences of other B-Lak genomes (Supplementary Table 4 and Supplementary Fig. 14). A subset of the reads (especially from B4, B7 and B9) probably derived from B-Lak genomes not reconstructed to date. In a few instances, adjacent SNP groups within individual Illumina reads directly indicate reassortment of alleles via homologous recombination (Supplementary Fig. 15).

The evidence of extensive homologous recombination motivated the question of whether phage relatedness (itself partially due to recombination) and phage admixture (sub-dominant genotypes within each population) could be predicted by baboon relatedness or social behaviour (Supplementary Table 5). Based on genetic (pedigree-based) estimates of kinship, grooming interactions (previously linked to similarities in baboon gut microbiomes, see Tungi et al.^16^) and host spatial proximity, there is no strong indication that these factors have strongly influenced the current phage genomes or within-population variation (Supplementary Fig. 16).

### Lak phages encode tRNAs with highly conserved introns

Interestingly, Lak phage genomes have tRNAs with introns. To our knowledge, introns in phage tRNAs have not been reported previously. Several occur in A1, A2 and C1 tRNAs and one occurs in tRNA Tyr (GTA) in the baboon Lak phage genomes (Table 1 and Supplementary Table 6). The set of tRNA introns in the A1, A2 and C1 genomes only partially overlap. Intriguingly, however, where the same intron occurs in the same tRNA, its sequence is typically identical across cohorts (Supplementary Information).

**Fig. 4:**
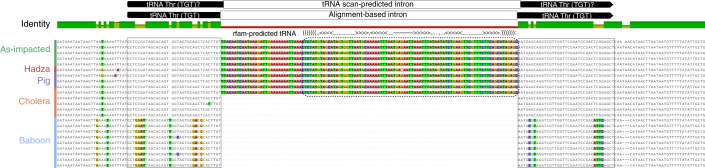
Alignment of sequences with a possible tRNA Thr (TGT). Some tRNAs are predicted to contain an intron that itself may encode a tRNA (dashed box with superimposed secondary structure). With the exception of the Hadza sequence, all introns are identical.

We predicted the tRNA intron sequences and found that a putative tRNA Thr (TGT) is predicted to encode a possible tRNA (Supplementary Table 6), the sequence of which is preserved perfectly in all but one case across cohorts. C1 and other phage fragments from the cholera cohort lack this tRNA intron. Alignment of sequences with and without this intron reveals that the intron is offset by one nucleotide compared to the predicted intron (Fig. 4).

### Lak phage metabolism and impact on host population dynamics

The vast majority of Lak protein coding genes are hypothetical (Supplementary Table 7). The largest inventory of genes with tentatively recognizable functions are involved in nucleotide, DNA and RNA transformations, functions previously noted as prominent in large phage genomes^9^. Phages may augment host translational machinery using sigma factors, translation initiation factors (for example, prokaryotic initiation factor-1), chain release factors as well as some genes that modify tRNAs (See Supplementary Information).

Given that a susceptible *Prevotella* host probably lacks CRISPR-based immunity, it is difficult to link Lak to the strain they are replicating in and thus to confidently infer the impact of phage predation on microbial community structure. Thus, we compared the abundances of all (rather than specific) *Prevotella* strains and Lak phage abundances over 4 time points for individuals 20 and 22. In both cases, phages are most abundant in the first sample and decline in abundance in the second sample, with corresponding increases in abundances of the more abundant *Prevotella* genotypes (Supplementary Fig. 17). *Prevotella* species probably differ to some extent in their metabolic capacities, growth rates and/or nutrient preferences; changes in relative abundances of species as well as their cumulative abundance could alter overall gut microbiome function. Based on the limited observations, shifts in *Prevotella* abundances due to phage predation may occur on the day-to-day timescale.

## Discussion

### Lak phages are common yet overlooked members of gut microbiomes

Another widespread phage recently discovered using metagenomics^21^, crAssphage, infects *Bacteroides*, bacteria typically associated with a Western diet. Lak phages infect *Prevotella* species, which are often abundant in the gut microbiomes of animals and humans consuming a high-fibre, low-fat diet^11^. Notably, the 15 complete curated Lak phage genomes are >5 times larger than the crAssphage phage genome^21^ and only ~4.6 times smaller than the genomes of their *Prevotella* hosts (Supplementary Information).

Why have megaphages that infect *Prevotella* been overlooked, and why are megaphages of any kind so rarely described? As suggested previously, very large phages are difficult to isolate due to restricted mobility on plates used for plaque assays^22^. Genome fragmentation hinders their detection via metagenomics. Further, phage structural proteins may be obscured by distant homology to known sequences and gene fragmentation when predicted using the wrong genetic code. Based on a meta-analysis of public data, Paez-Espino et al.^10^ produced a data set that included fragments classified as very large phage genomes. However, 11 of the sequences >200 kb are artefactual composites of identical repeated sequences (Supplementary Fig. 18). This underscores the importance of curation. Despite this, we suspect that many more phages with very large genomes will be uncovered in future metagenomic analyses.

### Environmental distribution and dispersal of Lak phages

Have Lak phages co-evolved with their hosts or has there been facile dispersal across animal habitat types? The Lak phages found in humans and baboons are less closely related than those found in humans and pigs (Supplementary Fig. 11). We did not detect patterns of *Prevotella* speciation consistent with animal host specificity (Supplementary Fig. 7), so we suspect that Lak phages as well as their bacterial hosts may be actively dispersing across animal habitats.

It was possible to probe the importance of homologous recombination in Lak phage evolution because multiple genomes were reconstructed from different baboons. The data clearly indicate extensive allele reassortment involving all of the analysed baboon phage populations. Presumably, recombination events require co-infection, that is, the coexistence of these huge genomes inside a *Prevotella* cell. We infer that recombination events are recent, based on the low frequencies of SNPs that distinguish otherwise identical sequence blocks in different B-Lak genomes, and we suspect it is ongoing, given the presence of minor recombinant variants within some populations. Overall, the results suggests that distinct phages were brought into contact relatively recently, possibly following migration from another animal reservoir. A similar phenomenon was previously reported in bacterial genotypes^23^. Consistent with the recent introduction of Lak phages is their prevalence in the baboon population and associated low level of CRISPR-based immunity.

If *Prevotella* and their megaphages migrate among animal and human microbiomes, they could carry with them genes that are relevant to human and animal health and the spread of disease. The concept of zoonotic viruses is well established, but there may be analogous phenomena involving phages. Phages can disseminate virulence factors between bacterial strains, including toxin-encoding genes responsible for many important diseases such as diphtheria, cholera, dysentery, botulism, food poisoning, staphylococcal scalded skin syndrome, necrotizing pneumonia or scarlet fever^8,24^ and propagate other genes of medical interest among animal reservoirs, such as those involved in antimicrobial resistance. The finding of related Lak phages in baboon, pig, cow and human populations suggests this possibility; the probability that it may occur is clearly increased where phages have huge genomes.

### Possible drivers of megaphage evolution

Interestingly, Lak phage genomes are in the size range of those of many putative bacterial and archaeal symbionts (for example, candidate phyla radiation bacteria and DPANN archaea (Diapherotrites, Parvarchaeota, Aenigmarchaeota, Nanoarchaeota, Nanohaloarchaea^25^)). Moreover, the nucleic acid-related functions predicted for Lak phage genes are similar to those predicted for genes of candidate phyla radiation bacteria^25^. Are jumbo phages and megaphages the consequence of random local genome expansion events, or might there be stabilizing forces that converge on a specific genome length? Because we generated complete genomes for phages from multiple distinct cohorts, we could document consistent genome sizes of ~540–552 kb, suggesting that evolutionary forces preserve large genome size. Particle size affects flocculation and attachment, and larger particles may be better retained in specific pore spaces in the gut environment compared to smaller particles. Clearly not all gut-associated phages are large, so at best, physical size can provide only a partial explanation.

The existence of megaphages motivates the general question of the costs and benefits to the phages of large genomes and the feedbacks that drive their evolution. Lak phage genomes encode many tRNAs, which could improve their replication success (see Supplementary Information), but the span of genome-encoding tRNAs is small. More probably, the hundreds of hypothetical proteins in the genomes may ensure successful phage replication in the face of host defence mechanisms and could also be important for increasing the host range.

Evolution of large phage genomes, and thus few expensive particles per replication cycle, could be an ecological strategy analogous to K- versus r-selection. Phages would normally be viewed as r-strategists, leveraging the advantage of many offspring to ensure high probability that a particle will find a host where it can replicate before loss of viability. For large phages, the countering trade-off of a shift towards K-selection could be improved survival as the result of the large capsid size. Potentially, this is because of the increased stability of larger capsids, for example, due to their smaller radius of curvature. Clearly, many factors could come into play, and direct experiments involving isolated phages and their hosts are required to understand the intriguing phenomenon of megaphages in human and other animal gut microbiomes.

## Conclusion

Megaphages are overlooked members of human and animal gut microbiomes. Their existence substantially increases the representation of phages whose genetic repertoires blur the boundaries that separate bacteria, bacterial symbionts and parasites/mobile elements. Their genomes hint at a fascinating biology and as yet unexplored complexity in the dynamics of gut microbiomes.

## Methods

### Samples, DNA and RNA extractions, sequencing and read archive analysis

Faecal samples were obtained from 10 Bangladeshi men (aged between 27 and 52 years) living in the Eruani village, Laksam Upazila, Bangladesh (samples were taken in April 2016). Informed consent was obtained from all individuals. All individuals displayed signs of arsenicosis and were consuming arsenic-contaminated drinking water. Samples were collected on 4 consecutive days (labelled days 2–5) and stored at −20 °C until they were shipped to the Santini Lab at University College London on dry ice. Samples were stored at −80 °C until nucleic acid extractions were performed. DNA was isolated with the PowerFecal DNA Isolation Kit (MO BIO Laboratories) according to the manufacturer’s instructions and stored at −20 °C. DNA samples were sent to RTLGenomics on dry ice and prepared for sequencing using the KAPA HyperPlus Kit (KAPA Biosystems) following the manufacturer’s protocol, except that the DNA was fragmented physically using a Bioruptor (Diagenode Diagnostics), instead of enzymatically. The resulting individual libraries were run on a Fragment Analyzer (Agilent) to assess the size distributions of the libraries, quantified using a Qubit 2.0 Fluorometer (Thermo Fisher Scientific), and also quantified using the KAPA Library Quantification Kit (KAPA Biosystems). Individual libraries were then pooled equimolar into their respective lanes and loaded onto an HiSeq 2500 (Illumina) 2 × 125 bp flow cell and sequenced. RNA was extracted from all 4 samples from individuals 20 and 22 with the PowerMicrobiome RNA Isolation Kit (MO BIO Laboratories) according to the manufacturer’s instructions and stored at −80 °C until they were shipped to the QB3 Center at the University of California Berkeley on dry ice for sequencing.

The cohort of cholera patients comprised 42 men, 3 women, and 2 male and 2 female children. These samples were previously sequenced; see Methods from David et al.^4^ for information regarding informed consent and sampling protocols. The original study was approved by the Ethical and Research Review Committees of the International Centre for Diarrhoeal Disease Research, Bangladesh and the Institutional Review Board of Massachusetts General Hospital.

The analysis of megaphages in pigs targeted samples in which faecal DNA from multiple pigs (*Sus domesticus*) on Danish pig farms were pooled before sequencing (*n* = 105 farms). Farm selection and sampling protocols were approved by the EFFORT consortium (Ecology from Farm to Fork Of microbial drug Resistance and Transmission). For details on study design (including randomization and blinding), see the Methods available from Munk et al.^26^.

The baboon cohort comprised 17 male adults and 31 female adults from two social groups. The baboons (*P. cynocephalus*) were a part of a long-term study tracking individual baboons from several social groups in the Amboseli ecosystem since 1971. Study design was approved by the Institutional Animal Care and Use Committee at Duke University (protocol no. A028–12–02) and Notre Dame (protocol no. 16–09–3339). For details regarding the Amboseli project, and the methods used for sample collection and processing for the baboon cohort, see the previously published study by Tung et al.^16^.

### Binning of draft genomes, genome curation and annotation

Bins were constructed from scaffolds of >1 kb in length based on the combination of genome guanine-cytosine content, coverage and a phylogenetic profile as described in Anantharaman et al.^27^. The phylogenetic profile was established based on gene-by-gene comparison to a reference genome data set^28^ We identified all sequences that encoded *ribosomal protein S3*, a gene that occurs in a relatively conserved block of genes that encode ribosomal proteins, and used these sequences to profile the overall community composition (taxonomy and abundance). Putative phage scaffolds were identified based on the high fraction of proteins with no related sequence in the database or similarity to phage proteins, as well as the presence of genes encoding structural proteins. Very large genome fragments were selected for curation. In cases where these were substantially shorter than the final genome length, candidate fragment collections identified based on consistency of guanine-cytosine content, coverage and phylogenetic profile were subjected to curation. Coverage values were determined by read mapping using Bowtie 2^29^ with default parameters for paired reads.

The first genome curation step involved identification of local assembly errors and either correction of the errors or gap insertion using ra2.py^30^. Curation of each genome was conducted independently and involved correction of local scaffolding errors and gaps, contig extension to enable joins and circularization, with manual resolution of regions of confusion. Reads from the sample were mapped to the scaffold assembled from that sample and unplaced paired reads used to extend ends and fill gaps. Curation was conducted in Geneious^31^. Regions of confusion were identified based on much longer than expected placement of paired reads or backwards mapping of paired reads. Reads were manually relocated and reoriented. The final curated sequences were visualized throughout to confirm complete and accurate coverage of each genome. Final genomes were checked to confirm the absence of large repeated sequences that could have confounded the assembly. The start position was chosen in a random region so as not to interrupt a gene. Later reconstructed genomes were adjusted so that the start positions corresponded to those of earlier assembled genomes.

Genes were predicted on scaffolds >1 kb using Prodigal^32^, initially using genetic code 11. Subsequently, Lak phage genes were re-predicted using code 15. Initial functional predictions were established based on similarity searches conducted using the basic local alignment search tool (BLAST) against the UniProt Knowledgebase and UniRef100 database^33^, and the Kyoto Encyclopedia of Genes and Genomes (KEGG)^34^ and uploaded to ggKbase (https://ggkbase.berkeley.edu/project_groups/megaphage). In addition, genes were annotated by scanning via hmmsearch^35^ with a collection of KEGG Hidden Markov Models representative of KEGG orthologous groups. The majority of phage structural genes were not identified in our initial functional predictions. Thus, we searched for proteins with unknown functions against the National Center for Biotechnology Information (NCBI) non-redundant protein database using position-specific iterative BLAST to identify remote homologues. These sequences were then aligned and searched against the Pfam_A_v31.0 and TIGRFAMs_v15.0 databases using HHpred to assign functions^36^. The amino acid sequences of terminases were so divergent from the terminases of previously analysed phages that they could not be identified using standard functional prediction methods. We found the terminase gene by searching all predicted phage proteins against a protein database of terminase large subunits, taken from phages spanning several different families and including all identified terminase large subunit proteins from phages with genomes >200 kb in length.

The tRNA genes were predicted using tRNA scan with bacterial settings^37^. tRNAs that were larger than expected and could not be assigned a classification were evaluated in terms of potential introns. Genes were re-predicted using the eukaryote settings (bacterial tRNA genes typically do not encode introns, so the program does not recognize them) to identify the tRNA type, anticodon and intron sequence. Intron excision points were re-evaluated based on the alignments of genes with and without inserted sequences. Intron sequences were tested for possible classification using rfam^38^.

Codon usage was calculated in consecutive 1 kb windows, and was reported as the percentage of a specific codon out of all codons in the coding region of that window. Calculations were done using a custom script, cu.py.

### CRISPR targeting analyses

CRISPR arrays were predicted on all scaffolds >1 kb in the Laksam Upazila cohort and the baboon cohort using a command line version of the program CRISPRDetect^39^ with parameter -array_quality_score_cutoff = 3. Only arrays with a score above the cut-off of 3 were considered. Spacers and repeat regions were extracted from the output files, and all spacers and repeats were searched against the Lak phage genomes A1 and A2 using BLASTn with the parameter -task = short. No repeat regions had a hit to A1 or A2, so all spacers with a hit containing ≤1 mismatches and a length >24 bp were considered to target Lak phages. The taxonomy of the scaffolds containing the CRISPR arrays with spacers targeting a Lak phage genome were determined by assigning taxonomy to all genes on the scaffold based on USEARCH clustering with the UniProt database. Scaffold taxonomy was assigned according to the highest taxonomic level shared by at least 50% of the genes on the scaffold. CRISPR arrays containing the repeat GGTTTAATCGTACCTTTATGGAATTGAAAT were chosen for reconstruction based on their high number of spacers targeting Lak phages. Arrays were manually aligned and spacers coloured using Geneious^31^.

### Testing for megaphages in other data sets

Bacteria of the genus *Prevotella* are abundant in the gut microbiomes of humans in the developing world. Thus, we wondered if related megaphages occur in other gut microbiomes that contain *Prevotella*. A search of NCBI’s non-redundant protein database for proteins related to those of the megaphages yielded no significant hits, so we selected individual metagenomic data sets from *Prevotella-*enriched samples for deeper analysis. Read data sets from previously published studies were selected based on the sampled environment and information about *Prevotella* content and downloaded from the NCBI’s sequence read archive. Reads were mapped to the Lak phage genomes initially assembled from the Laksam Upazila cohort to determine whether or not Lak phages were present in the sample. Selected read sets were trimmed using sickle with default parameters (https://github.com/najoshi/sickle) and each data set was assembled separately using IDBA-UD^30^ with default parameters.

### Phylogenetic and community compositional analyses

Community composition (Supplementary Fig. 1 and Supplementary Fig. 5) was determined by read mapping to the conserved *ribosomal protein S*3 gene (*RPS3*). The *RPS3* genes were identified on all scaffolds >1 kb in the Laksam Upazila Bangladeshi cohort and the baboon cohort, and classified to the species level based on USEARCH clustering^40^ with annotated proteins in the UniProt database. All *RPS3* genes were then clustered at 90% identity using USEARCH, and a representative sequence from each cluster was chosen. Reads from each sample (all 10 people, 3 or 4 samples from consecutive days per person for the Bangladesh cohort and all 48 baboons) were mapped to these representative sequences, and the percentage coverage of each *RPS3* gene was determined. Percentage coverage was then normalized by the sequencing depth of each sample to determine percentage project. Any genus that was present in <10% cumulative abundance across all samples was grouped into the ‘other’ category. The stacked bar charts in Supplementary Figs. 1 and 5 were generated by plotting the percentage project of each genotypic variant in the same order for each sample, sorted by assigned genus. The bars were then coloured by genus, resulting in coloured genus bars divided by the genotypic variants within that genus (grey lines). The figure was plotted using the Matplotlib Python library^41^.

The *Prevotella* phylogenetic tree was constructed using 16S rRNA gene sequences. First, the Greengenes database^42^ of complete 16S rRNA gene sequences was augmented with all 16S rRNA gene sequences from *Prevotella* reference sequences on the NCBI that were independently classified as *Prevotella* (15 were assigned to a genus other than *Prevotella* and discarded). This augmented database was then used to classify 16S rRNA gene sequences from all samples in each study where a megaphage was found, including samples in publicly available studies, using the assign_taxonomy.py script from qiime1 and default parameters^43^. Sequences classified as *Prevotella* were aligned with all known reference *Prevotella* 16S rRNA gene sequences and an *Escherichia coli* 16S rRNA gene outgroup (NCBI ref. J01859.1) using MUSCLE^44^. A tree was generated using RAxML-HPC2 on XSEDE^45^ on the CIPRES Science Gateway^46^ using parameters raxmlHPC-HYBRID -T 4 -n result -s infile.txt -m GTRGAMMA -p 12345 -k -f a -N 100 -x 12345 --asc-corr lewis. The tree was edited and annotated with iTOL^47^.

### Comparative genomics

Genome sequences were aligned using the progressiveMauve algorithm using default parameters^48^. In certain regions, the sequences were offset because the algorithm failed to align them. In some cases, this could be corrected based on visual inspection. In other cases, the sequences were superimposed to constrain the overall alignment length; very low similarity scores were then displayed.

Predicted proteins from all known Lak phage genomes or bins were clustered along with predicted proteins from jumbo *Sphingomonas* phage PAU (ref: NC_019521.1) using MMseqs^49^. Fifteen protein subfamilies were identified that contained at least one protein from all Lak phage genomes or bins used. The terminase large subunit protein from phage PAU was clustered into a different subfamily from the Lak terminase large subunit proteins, and those two subfamilies were combined to generate 16 protein subfamilies used in further analysis. All subfamilies were manually curated to ensure only one protein from each Lak phage genome or bin or PAU was included. (In all cases where more than one protein from the same Lak phage genome or bin were grouped into the same subfamily, only the protein with the highest percentage identity to the rest of the subfamily was retained.) Proteins within each family were aligned using MUSCLE; alignments were concatenated according to the order the genes appeared in the A1 genome using Geneious^31^. A tree was generated using RAxML-HPC2 on XSEDE^45^ on the CIPRES Science Gateway^46^ using parameters raxmlHPC-HYBRID -T 4 -n result -s infile.txt -p 12345 -m PROTGAMMADAYHOFF -f a -N 100 -x 12345 --asc-corr lewis. The tree was rooted using the concatenation derived from *Sphingomonas* phage PAU. The tree was edited and annotated with iTol^47^.

### Pig genome fragment analysis

To search the 105 metagenomes constructed from collections of faecal samples from Danish pig farms, we aligned all the assembled pig metagenomic scaffolds >1 kb in length against the Lak phage A1 reference genome using NUCmer (Mummer version 4.0.0beta). Filtering was done, so that alignments of at least 2 kb and 70% nucleotide identity were kept. The lengths of scaffolds meeting these criteria were summed to estimate the total genome sequence attributable to Lak phages, and the total alignment length was calculated. A metagenome was considered to contain Lak phages as long as at least one scaffold with an alignment length >2 kb was identified.

### Statement of ethics

The human faecal samples obtained were part of a clinical phase I/II study in rural Bangladesh entitled ‘Selenium and arsenic pharmacodynamics’ (SEASP) run by Graham George (University of Saskatchewan) and funded by the Canadian Federal Government, through a programme entitled Grand Challenges Canada-Stars in Global Health, with additional funds from the Global Institute for Water Security at the University of Saskatchewan. The SEASP trial was approved by the University of Saskatchewan Research Ethics Board (14–284) and the Bangladesh Medical Research Council (940,BMRC/NREC/2010-2013/291). Additional ethics approval was also obtained by UCL (7591/001).

### Reporting Summary

Further information on research design is available in the Nature Research Reporting Summary linked to this article.

## Supplementary information

Supplementary Information Supplementary Discussion, Supplementary Figures 1–18, Supplementary Table legends and Supplementary References.

Reporting Summary

Supplementary File 1 Laksam CRISPR arrays: CRISPR arrays with spacers targeting megaphage genomes from the Laksam Upazila cohort.

Supplementary Tables Supplementary Tables 1–7.

## Data Availability

The cu.py script is available at https://github.com/oddaud/cu.py.

## Data availability

The 15 Lak phage genomes have been deposited at NCBI under BioProject PRJNA491720. The genomes can also be downloaded from https://ggkbase.berkeley.edu/project_groups/megaphage. The read archive and other accession information is provided in Supplementary Table 1. Please note that it is necessary to register for a ggKbase account by providing an email address before accessing or downloading the data.
