# Supplementary material for: Megaphages infect Prevotella and variants are widespread in gut microbiomes
Source: Nat Microbiol. 2019 Jan 28;4(4):693–700. doi: 10.1038/s41564-018-0338-9 (PMC6784885; doi:10.1038/s41564-018-0338-9)
Supplement: Supplementary file 1 — Supplementary Discussion, Supplementary Figures 1–18, Supplementary Table legends and Supplementary References. [file 41564_2018_338_MOESM1_ESM.pdf]

nature microbiology

SUPPLEMENTARY INFORMATION

https://doi.org/10.1038/s41564-018-0338-9

In the format provided by the authors and unedited.

Megaphages infect *Prevotella* and variants are widespread in gut microbiomes

Audra E. Devoto[1], Joanne M. Santini[2], Matthew R. Olm[3], Karthik Anantharaman[1,12], Patrick Munk[4], Jenny Tung[5], Elizabeth A. Archie[6], Peter J. Turnbaugh[7,8], Kimberley D. Seed[3,8], Ran Blekhman[9], Frank M. Aarestrup[4], Brian C. Thomas[1] and Jillian F. Banfield[1,10,11]*

[1]Department of Earth and Planetary Science, University of California, Berkeley, Berkeley, CA, USA. [2]Institute of Structural & Molecular Biology, Division of Biosciences, University College London, London, UK. [3]Department of Plant and Microbial Biology, University of California, Berkeley, Berkeley, CA, USA. [4]National Food Institute, Technical University of Denmark, Lyngby, Kongens, Denmark. [5]Department of Evolutionary Anthropology, Duke University, Durham, NC, USA. [6]Department of Biological Sciences, University of Notre Dame, Notre Dame, IN, USA. [7]Department of Microbiology and Immunology, University of California, San Francisco, San Francisco, CA, USA. [8]Chan Zuckerberg Biohub, San Francisco, CA, USA. [9]Department of Genetics, Cell Biology, and Development, University of Minnesota, Minneapolis, MN, USA. [10]The University of Melbourne, Melbourne, Victoria, Australia. [11]Innovative Genomics Institute, University of California, Berkeley, Berkeley, CA, USA. [12]Present address: Department of Bacteriology, University of Wisconsin–Madison, Madison, WI, USA. *e-mail: jbanfield@berkeley.edu

Supplementary Discussion

Identification of genomes as phage-derived

Three possibilities were considered for the identity of the reconstructed complete genomes referred to here as Lak megaphage. First, given the greater than ~540 Kbp circular chromosomes, they could be small bacterial or archaeal genomes. This possibility is ruled out given the complete lack of ribosomal proteins and set of tRNA synthetases, among other components required for life. Second, they could be plasmids or phage. Arguing against the plasmid identification is the absence of typical plasmid genes, including origin of replication / replication initiation protein, partition genes etc. The first argument in support of phage identification is the presence of expected phage proteins, including terminase, portal vertex, baseplate wedge, tape measure, tail-related proteins etc. (see Supplementary Table 7). These are in generally conserved order over the chromosomes of Lak from multiple cohorts, arguing against random acquisition of phage structural genes by a plasmid. These phage proteins are not co-located within each genome, as occurs in most small phage genomes, but this is not uncommon in large phage genomes. The genomes also encode many other proteins (~6%) most closely related to proteins from other phage, as well as a large number of hypothetical proteins with no known matches to any database. Second, the ratio of abundance of the phage chromosomes compared to the abundance of most abundant Prevotella chromosomes is >12:1 in three of the Baboon samples. At least 12 copies of a >540 kbp phage genome (>6.5 Mbp of sequence) in a Prevotella cell with a genome of ~ 2.5 Mbp seems an extraordinary claim, and unlikely. Further, in samples collected over consecutive days the ratio of Lak : all potential *Prevotella* hosts changes dramatically (i.e., no abundances are correlated), a phenomenon not anticipated for a plasmid but common in the event of phage blooms (Supplementary Figure 6). For example, the abundance ratio for Lak to most abundant *Prevotella* changes from ~12:1 to ~0.5:1 over three days (individual 22, source of the A1 genome). Finally, we identified endolysin genes in all Lak genomes. This enzyme degrades the bacterial cell wall, and would not be required by plasmids. Overall, the evidence conclusively supports the determination that these genomes derive from phage.

No evidence for megaphage as prophage

No reads or read pairs connected the megaphage scaffolds to bacterial scaffolds, and no phage fragments were found in bacterial scaffolds in the newly generated dataset. We checked all NCBI *Prevotella* genomes for fragments of the phage using MUMmer (1). The only finding was a ~60 bp region identical in several *Prevotella* genomes and A1. This small region occurred at the end of a transposon in *Prevotella* and in an intergenic region in the phage genome. Thus, to date there is no evidence that the megaphage integrate into *Prevotella* genomes. For the 44 *Prevotella* genomes reconstructed from the baboon datasets, the median size was 2.38 Mbp (based on the cumulative length of scaffolds assigned to *Prevotella* genome bins), with a median of 50/51 expected single copy genes and 0.5 duplicated single copy genes per genome. For examples, see: https://ggkbase.berkeley.edu/project_groups/megaphage.

Detection of phage in other subjects of the Laksam Upazila cohort

In Subject 21, co-assembly of reads mapping to phage A1 from all four samples resulted in 195,292 bp of contiguous sequences, the largest of which were around 900 bp in length and comprised of perfectly mapping reads. There was also read mapping across the entire A1 genome from samples

from Subject 23, but the coverage was too low for assembly. All other samples had fewer than 250 reads mapping non-specifically to the A1 genome.

Identification of the megaphage host as Prevotella

We extracted spacer sequences from all 2,485 CRISPR loci encoded on reconstructed genome fragments of >1000 bp in length. From 26 loci, 12 of which were in genomes of *Prevotella* species and 14 of which were on scaffolds too short to assign taxonomy to, 46 unique spacers targeted the A1 and A2 genomes. In many cases, spacers targeted both genomes, as expected given their high overall nucleotide similarity. Most of the Cas systems are Type I or Type II (with Cpf1, or Cas9) and spacers from both systems target the megaphage. Loci from these systems are known to undergo rapid expansion, and in some cases the *Prevotella* arrays contain up to 161 spacer repeat units. Most CRISPR loci show the typical pattern of shared spacers at one end and non-clonality associated with the currently diversifying CRISPR locus end.

Only six (of 38) samples lacked *Prevotella* with CRISPR spacers that target the A1 and A2 phage, two from Subject 22 and all four samples from Subject 20. The lack of CRISPR spacer-based immunity may explain why the phage proliferated in those microbiomes. In fact, *Prevotella* species with spacers that target the megaphage occurred in samples from all subjects from which the phage was not reconstructed, consistent with the expectation that perfect spacer targets preclude infection at any appreciable level. Based on the prevalence of megaphage-targeting spacers, we conclude that these megaphage are common in the microbiomes of this human cohort.

To our knowledge, no phage larger than 200kb have been reported to infect *Prevotella.* Most jumbo phage hosts known so far are also gram negative, however, such as *Synechococcus, Pseudomonas, Caulobacter, Vibrio, Erwinia,* and *Aeromonas (2)*.

Phage isolation attempts

We attempted to isolate the phage using faecal samples from subjects 20 and 22 following a similar method described previously (3). *Prevotella copri* (DSM 18206^{T} was used as the host and grown in liquid and on solid media as described previously (4). In brief, faecal samples (about 1 g) were suspended in 1 ml buffer, vortexed, centrifuged to remove particulate material and filtered through a 0.45 µm filter. Confirmation that the Lak phage was present in the extract was done by PCR using Lak-specific primers. Mid-exponential phase *P. copri* cultures were used to inoculate plates (4) which were then seeded with the phage extract, incubated and then visualised for cell lysis. As was the case in the prior crAssphage study, isolation was not achieved, which may be due to a number of reasons including that *P. copri* is not a Lak phage host.

tRNA analysis

We compared the sets of tRNAs in the baboon megaphage genomes and found that they are more similar to each other than to the sets in the other megaphage genomes (Supplementary Table 2A), although allowing for rearrangements, there are strong similarities in the tRNA complement across megaphage from all datasets (Supplementary Table 2B).

Questionable tRNA are recognized when the isotype predicted based on the overall tRNA sequence alignment does not match that predicted by the anticodon. In the case of Sup tRNA a mutation has occurred in the anticodon so that it now matches a stop codon. The tRNA scan program predicted glutamine as the third most likely amino acid linked to the Sup CTA tRNA, but this prediction is unreliable because the phage sequences are far distant from those used to build the models (Todd Lowe, pers. comm). The relevance of the other Sup tRNAs is uncertain.

Phage-encoded tRNAs may correspond to codons that are abundant in certain phage genes and thus increase their translation efficiency (5). They may also decrease the probability of mismatches between host and phage codon preference and thus increase host range (2). The tRNAs and pseudo-tRNAs may have other purposes, however. For example, they may confuse the translational apparatus through loading of the wrong amino acid or stall translation due to inability to load amino acids.

Detection of Lak phage in other Baboon metagenomes

Partial genomes were identified in the following Baboon metagenomic datasets:

1747022 (M05) ~540 kbp in six scaffolds, largest is 230 kbp.
1747029 (M06) ~537 kbp in three scaffolds, largest is 432 kbp.
1747031 (M08) ~546 kbp in two scaffolds, larest is 361 kbp
1747033 (F07) ~545 kbp in five fragments, largest is 281 kbp.
1747038 (F12) ~546 kbp in two scaffolds, largest is 361 kbp.
1747039 (M10) ~ 541kbp in four scaffolds,largest is 151 kbp.
1747060 (F28) ~ 546 kbp in two scaffolds, largest is 361 kbp.

Subsequent to this study an additional B-Lak genome (B10) was curated to completion and is available for analysis.

Bins are available: https://ggkbase.berkeley.edu/project_groups/megaphage

Please see "Data Accessibility" section for details.

B-Lak population variation analysis

As documented in Supplementary Table 4, the B1, B2, B3 and B5 populations are relatively clonal, and ~96.4% of reads that mapped to the genome using standard parameters can be mapped with zero SNPs. For these phage, 0.01% - 0.02% of the reads map perfectly to another B-Lak genome. The B6 and B8 populations are only slightly less clonal and show similar fractions of reads with perfect matches to other B-Lak genomes (0.01 and 0.4% of all reads). Even for the other populations, B4, B7 and B9, between 94% and 94.7% of reads match perfectly to the reconstructed sequence, again providing confidence that the reported genomes are not chimeras of population variants.

Population datasets for B4, B6 and B9 provide the best evidence for the presence of sub-dominant sequence types characteristic of another B-Lak population (probably as recombinant genotypes, given the high degree of localization of the read mappings). More than ~0.1% of reads from some population datasets map to other genomes (B4 reads to the B2, B8 and B9 genomes; B6 reads to the B3 and B9

genomes and 9 reads to five other genomes). The B9 genome is notable in that it was reconstructed from five genome fragments that comprised ~99% of the final bin length. Fragmentation was at least partially due to a few local regions of within-population sequence variation.

tRNA intron similarity across cohorts

Introns were identified within some pseudo tRNAs that are substantially larger than expected. An identical tRNA Phe (GAA) intron is found in A1, A2, the cholera cohort and some pigs; identical introns occur in a tRNA Gly (TCC) in the A2, C1 and some fragments from pigs and identical tRNA Thr (TGT) introns occur in A1, A2, C1 and some genome fragments from the cholera and pig cohorts. Finally, we identified identical introns in Tyr (GTA) from A1, A2 and C1 and related but longer introns in the B-Lak phage genomes (see Supplementary Table 6).

The largest putatively circularized phage genome reported to date from any environment is 595,573 bp in length, with an unknown host (6). That genome is predicted to encode 75 tRNAs, 44 of which have unknown or mismatch isotypes. We predict that this genome has at least 4 tRNAs with introns. Thus, tRNAs with introns may be fairly common in large phage genomes.

Phage metabolic potential

The largest fraction of genes with confident functional predictions are involved in reactions involving nucleotides (see Supplementary Table 7). Some genes encode enzymes that interconvert various deoxyribonucleoside and ribonucleosides, phosphorylate/dephosphorylate nucleotides and participate in other steps in DNA and RNA metabolism (e.g., ribose-5P to PRPP). In addition, there are helicases, topoisomerases, polymerase subunits, primases, exonucleases, genes involved in restriction, base excision and repair and recombination (potentially of relevance given the extensive evidence for homologous recombination). Similar sets of genes have been reported in other jumbo phage, such as *Ralstonia* phage ϕRSL1 (7), *Xanthomonas* phage XacN1 (8), and *Cronobacter* phage GAP32 (9).

The Lak genomes encode several genes involved in carbon metabolism and genes involved in interconversion of nicotinate and nicotinamide, possibly with an impact on host energy metabolism. Also predicted is pnuC, a nicotinamide mononucleotide transporter. Other genes involved in transport include a co-located three subunit NitT/TauT (sulfonate/nitrate/taurine) system. The genomes also typically encode multiple molecular chaperones. Repeat sequence analysis identified a few genes with similar amino acid sequences that were probably duplicated.

Intriguingly, the genomes encode one or two proteins predicted to hydrolyze a compound related to nucleoside 3',5'-cyclic phosphate to nucleoside 5'-phosphate. Such enzymes might affect the cellular levels of the host's cyclic second messengers. Potentially related, the genomes also encode a T4-encoded RNA ligase, which phage may use to repair tRNAs that are broken when a host defense mechanism produces 2′,3′-cyclic phosphate ends (10)

Among the enigmatic predicted proteins of potential significance from the perspective of host metabolism is an enzyme annotated as heme oxygenase [EC:1.14.99.3], which catalyzes the degradation of heme, an iron complex outer membrane receptor protein and a glycosyltransferase implicated in lipopolysaccharide biosynthesis. Despite the genome size however, we could recognize

relatively few genes that might supplement host metabolism, except as it might pertain to phage replication.

Interestingly, the genomes encode some genes that modify tRNAs, such as for adding a guanine nucleotide to the 5′ end of $tRNA^{His}$, peptidyl-tRNA release, and CCA addition for tRNA maturation.

Other possible sightings of the Lak phage

A huge *Prevotella* phage from the cow rumen was imaged previously (11). The imaged phage was reported to have an icosahedral head of ~120 nm in diameter and a long curved tail that was 30 nm wide and up to 800 nm long. To our knowledge, the largest isolated phage infects *Bacillus megaterium* and has a 497 kbp genome, a head diameter of 160 nm and tail length of 453 nm (2). The visualized phage may be related to Lak megaphage.

Supplementary Figures

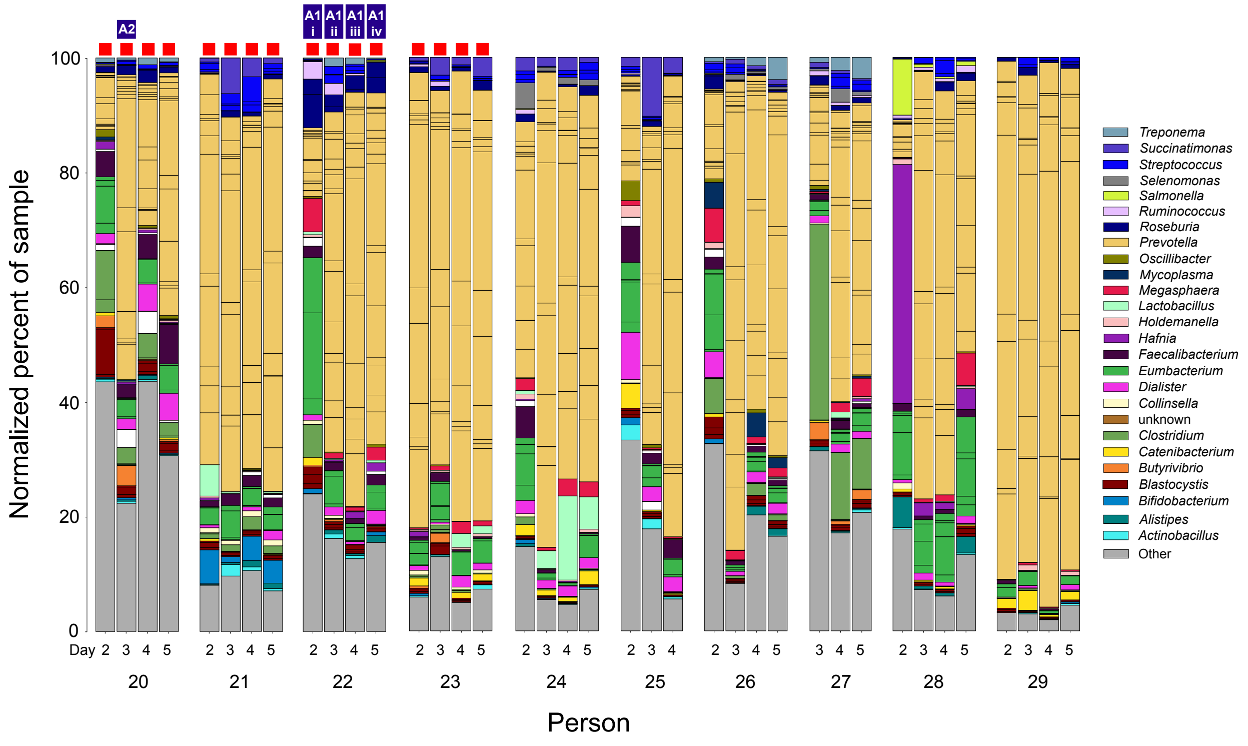

Supplementary Figure 1: Community composition of gut microbiomes of ten subjects from Laksam Upazila, based on phylogenetic analysis of ribosomal protein S3 sequences. Each stacked bar represents one genotypic variant. Variants are in conserved order and colored by genus. All genotypic variants present in under 10% abundance across all samples are collapsed into the “Other” bar. The increments in the stacked bar chart are colored by genus. Red boxes indicate samples from which megaphage sequences were partially assembled; blue boxes indicate samples from which a complete megaphage genome was recovered. Lines within each genus denote the relative abundance of genotypic variants. The high variability seen in day 2 samples compared to day 3, 4, and 5 in some subjects is confirmed by 16S amplicon sequencing and likely a result of a change in diet upon hospital admittance.

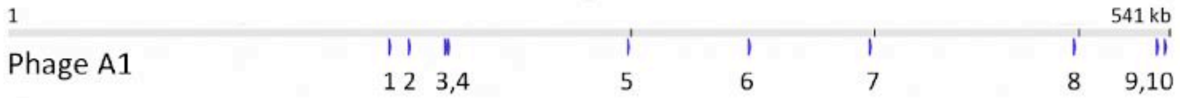

	Position	Form of variation	Genomic site	A1_22-2	A1_22-3	A1_22-4	A1_22-5
1	178658	AATAA insert	Intergenic	0.048	0.093	0.044	0.048
2	187839	G vs. A	Intergenic	0.333	0.536	0.342	0.706
3	204732	T vs. C	Hypothetical protein: K/R	0.485	0.565	0.427	0.389
4	206225	G vs. T	Hypothetical protein: M/I	0.486	0.483	0.42	0.412
5	290461	ATC vs. CTT	Hypothetical protein: D/K	0.368	0.466	0.58	0.667
6	346200	G vs. T	Hypothetical protein: V/L	0.471	0.556	0.302	0.389
7	403206	ATAAAA insert	Hypothetical protein: insert FI	0.2	0.224	0.252	0.097
8	498223	ATATTATTTTTTAA insert	Hypothetical protein: extends end by FKK	0.234	0.088	0.056	0.125
9	536679	add T in hypervariable T region	Intergenic	0.098	0.146	0.192	0.2
10	540264	C vs. T	Hypothetical protein: G/D	0.458	0.483	0.47	0.519

0 → 1

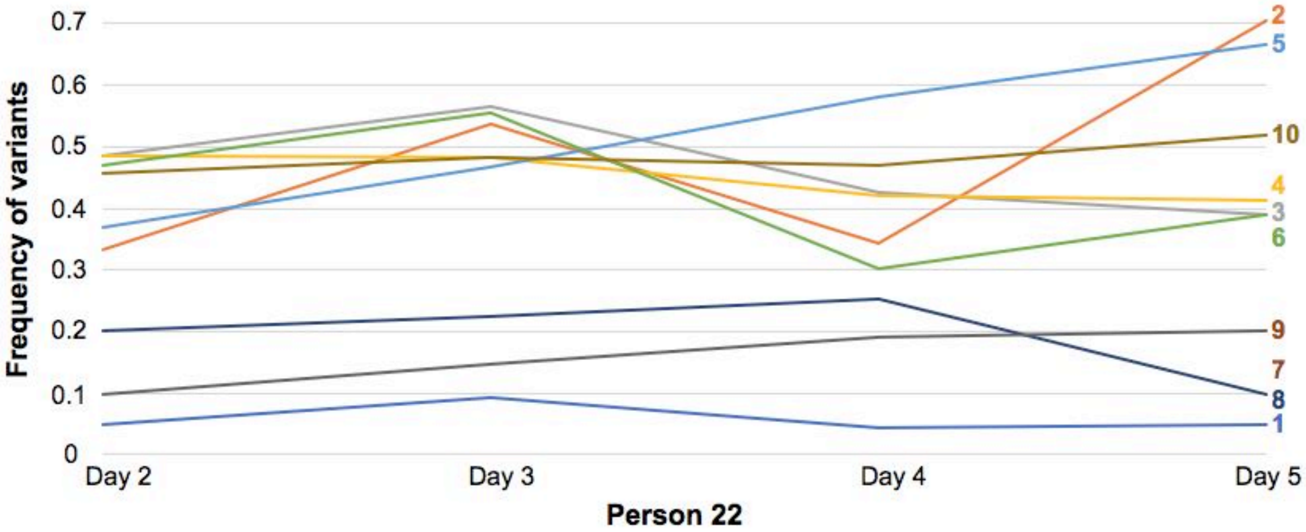

Supplementary Figure 2: Analysis of the frequency of variants at ten variable sites (numbered blue tick marks) in the genomes of the Lak phage A1 in samples collected over four sequential days (22-2, 3, 4, 5). The table lists the variant site types, consequences of variation and site frequencies. The graph shows how the relative abundance of each variant changed over the sample series. Differences in the frequencies of variants at each site in a single dataset in combination with divergent patterns of variant frequencies over the sample series suggest that few if any of the variants are linked.

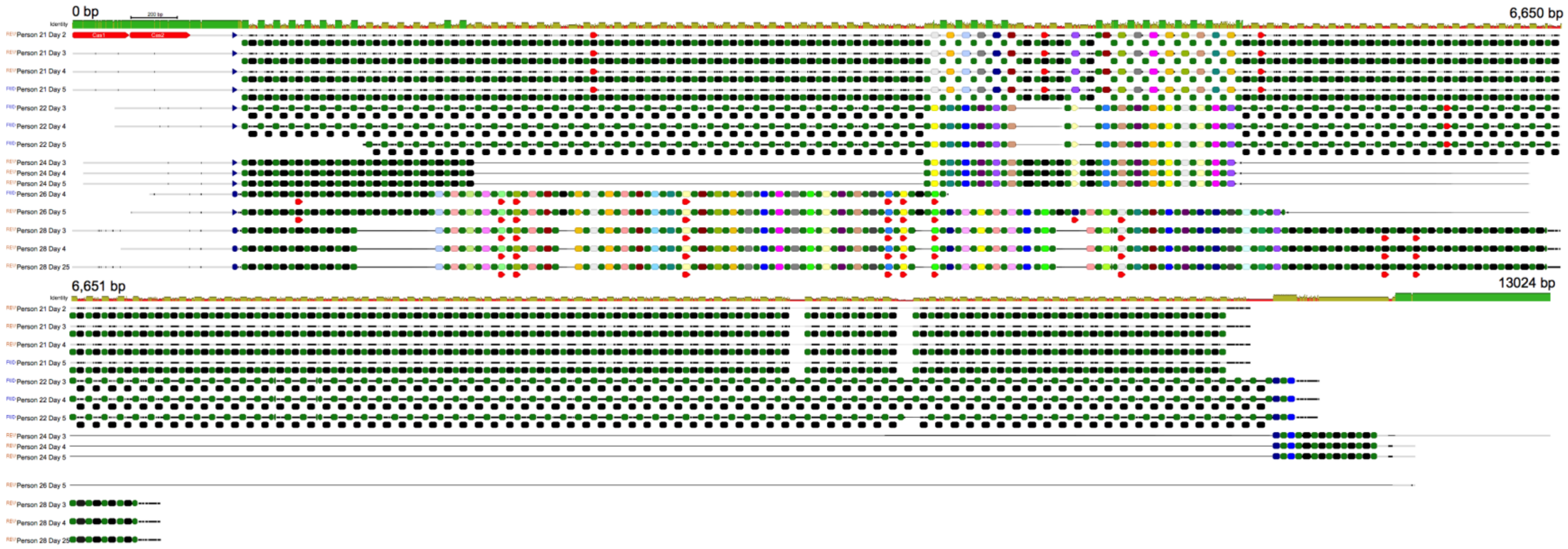

Supplementary Figure 3: Full reconstruction of CRISPR arrays targeting megaphage from subjects 21, 22, 24, 26, and 28. Note that the *Prevotella* arrays from sample 22, where the phage is abundant and was first identified, has only one spacer targeting the megaphage. For details and color legend, see **Figure 1**.

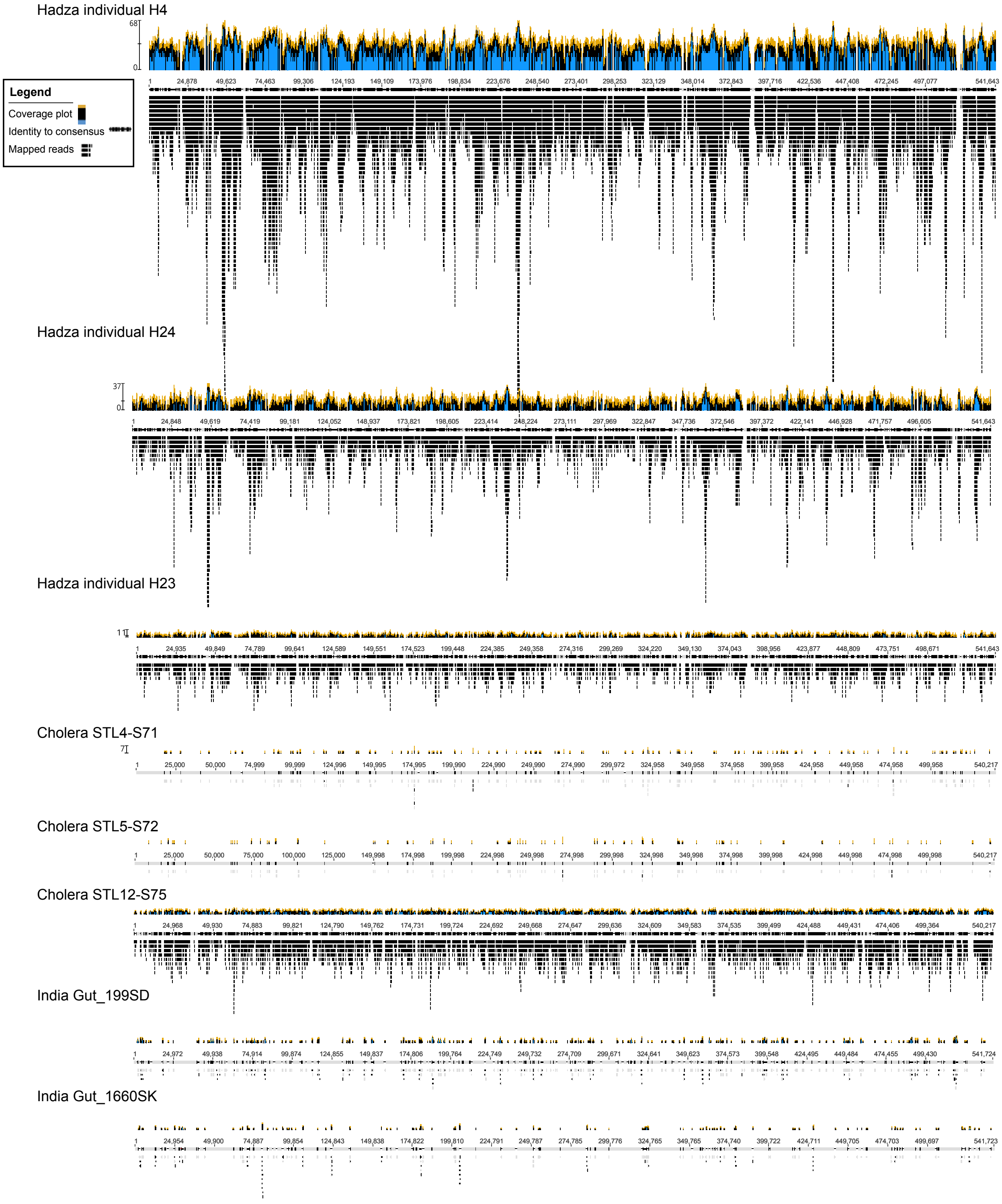

Supplementary Figure 4: Overview of reads mapped to the Lak phage A1 genome from various cohorts. The coverage scale (in blue) is approximately even throughout the presentation. For data accession numbers, see **Supplementary Table 1**. Data sources are as follows: Hadza, (12); Cholera, (13); India, (14).

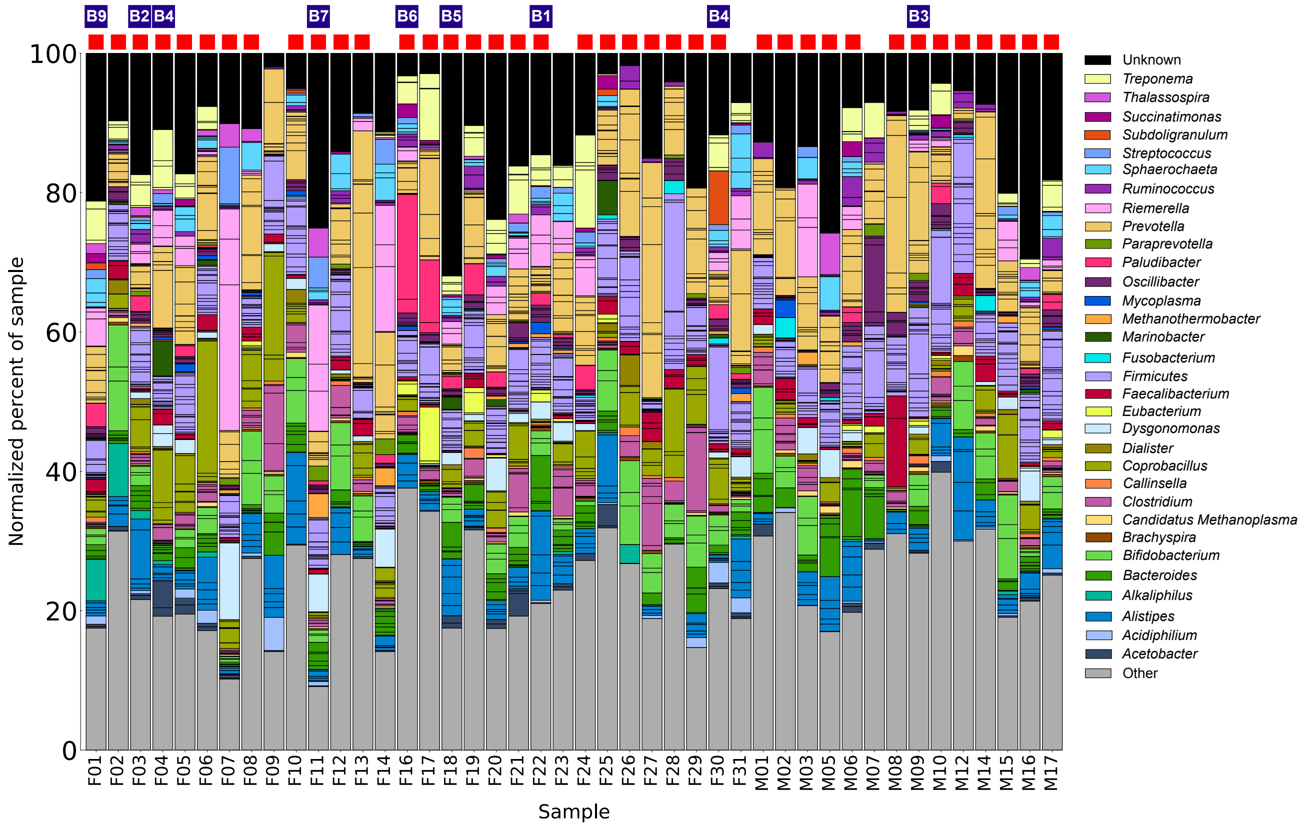

Supplementary Figure 5: Overview of the community composition of baboon faecal samples. Red squares indicate samples from which Lak phage were partially assembled, blue squares indicate samples from which complete Lak genomes were reconstructed. Baboon sequencing data first reported by (15).

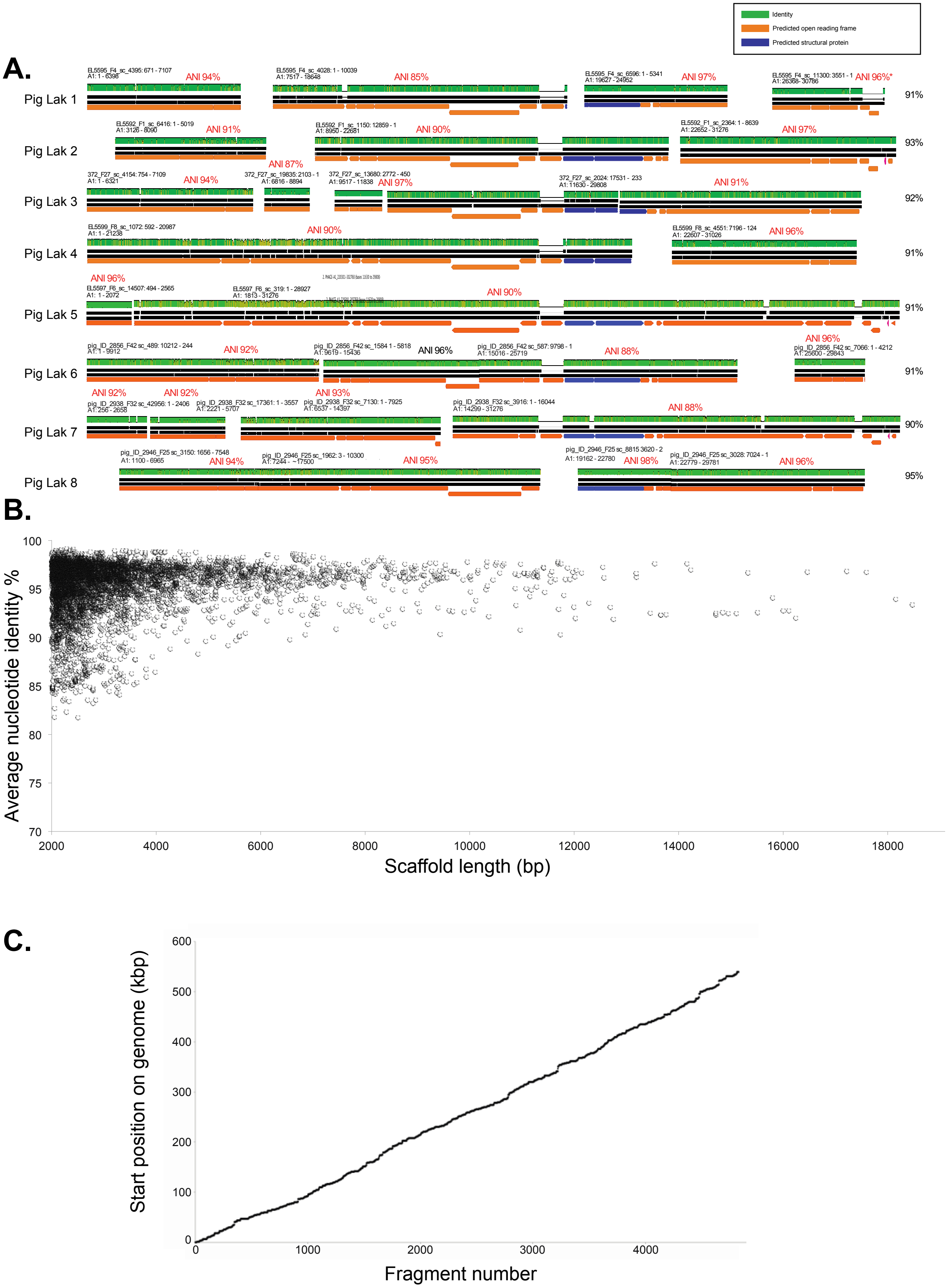

A.
Identity
Predicted open reading frame
Predicted structural protein
Pig Lak 1
Pig Lak 2
Pig Lak 3
Pig Lak 4
Pig Lak 5
Pig Lak 6
Pig Lak 7
Pig Lak 8
91%
93%
92%
91%
91%
91%
90%
95%
B.
Average nucleotide identity %
Scaffold length (bp)
C.
Start position on genome (kbp)
Fragment number

Supplementary Figure 6: Fragments assembled from pig farm metagenome datasets were identified as deriving from Lak phage based on their sequence identity to the A1 Lak phage genome. The figure shows eight examples. Given that most fragments were <10 kbp in length, we mapped fragments to a ~30 kbp region of the A1 genome (within that shown in Figure 2) and identified cases where the fragments spanned >70%. The ANI for each fragment is shown above the fragment in red and the ANI for the set of aligned fragments is shown to the right. Over the 8 examples, the average ANI is 91.8 ± 1.7%. The average length of aligned scaffolds to the ~30 kbp A1 genomic region is 27.2 ± 2.6 kbp (the sum of the aligned scaffolds is slightly longer in some cases). Mapping of reads to the scaffolds from Pig Lak 8 confirmed a high degree of within-population sequence variation that likely accounts for assembly fragmentation. **B.** Plot of ANI vs. fragment length for all (N=4830) pig fragments >2 kbp identified as possible Lak phage. **C**. Plot of fragment number (N=4830, same fragments as in **B**) vs. start position on genome.

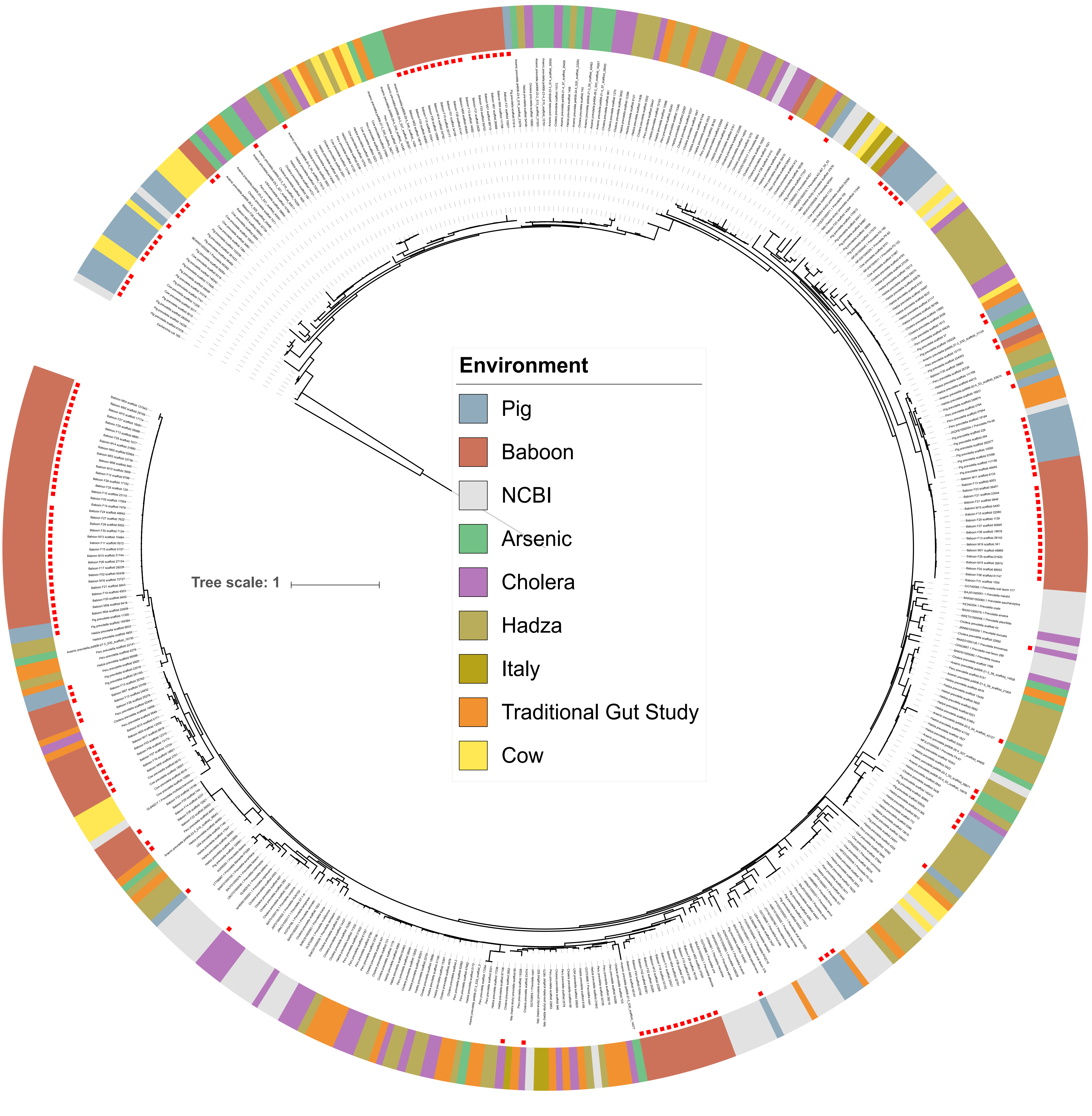

Environment
Pig
Baboon
NCBI
Arsenic
Cholera
Hadza
Italy
Traditional Gut Study
Cow
Tree scale: 1

Supplementary Figure 7: Phylogenetic tree of *Prevotella* from all cohorts examined in this study, constructed using 16S rRNA gene sequences on scaffolds >1 kbp in length, with information about the sample of origin. Branch lengths represent nucleotide substitutions per site. Red dots denote *Prevotella* strains that came from samples where Lak phage were also found. Note there is no strong separation of *Prevotella* type based on sample source and closely related strains come from samples with and without the megaphage. Data sources are as follows: Traditional gut study: (16), Baboon: (15), Hadza and Italy: (12), Cholera: (13), Cow: (17) and Pig data (this study). All data accession numbers listed in **Supplementary Table 1**.

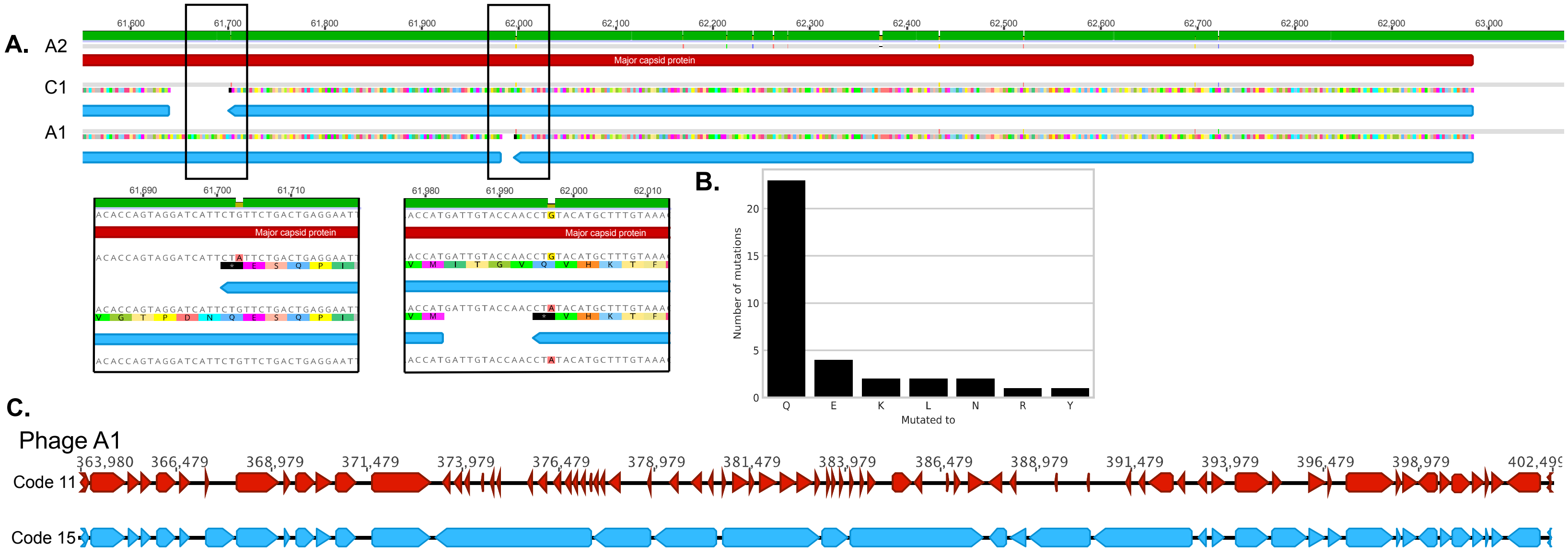

Supplementary Figure 8: Evidence for alternative coding involving the stop codon TAG translated as glutamine (Q). **A**. An example of TAG mutated to an alternative codon encoding glutamine in A1, A2, and C1. **B**. Number of times across the three genomes that TAG is mutated to encode an amino acid. **C.** Gene predictions using genetic code 15 (blue open reading frames) *vs.* 11 (red open reading frames). Note some genes are not affected by the change from the prediction of TAG as a stop codon to encoding glutamine, whereas others are. Use of the alternative code corrected for clearly split genes and increased the predicted coding density from <70% to ~90%, genome wide.

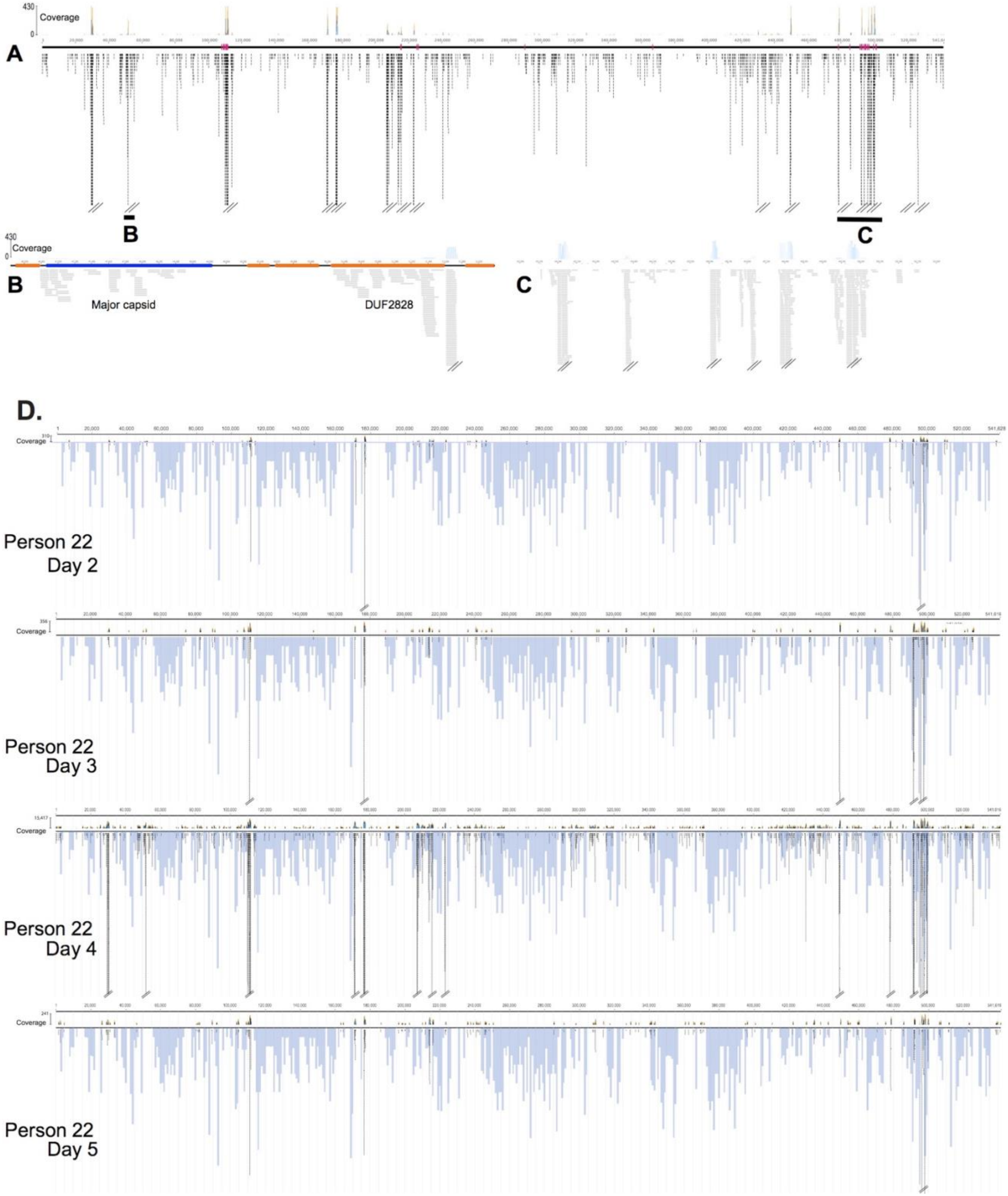
A
B
C
D.
Coverage
Major capsid
DUF2828
Person 22
Day 2
Person 22
Day 3
Person 22
Day 4
Person 22
Day 5

Supplementary Figure 9: Mapping of transcript reads to the A1 megaphage genomes. **A.** Reads from 22-4. Red tick marks indicate the locations of tRNAs. Paired lines indicate read pile ups that have been truncated to save space (see coverage information in the top graph). Heavy black underlines indicate regions expanded in **B**. and **C**. Note the high level of expression of many intergenic regions, some expression of tRNAs and of certain proteins. **D**. Mapping of transcripts to the A1 genomes (as in A-C) superimposed on histograms showing the TAG use frequency in blue. In all cases, regions with and without the TAG codon are expressed.

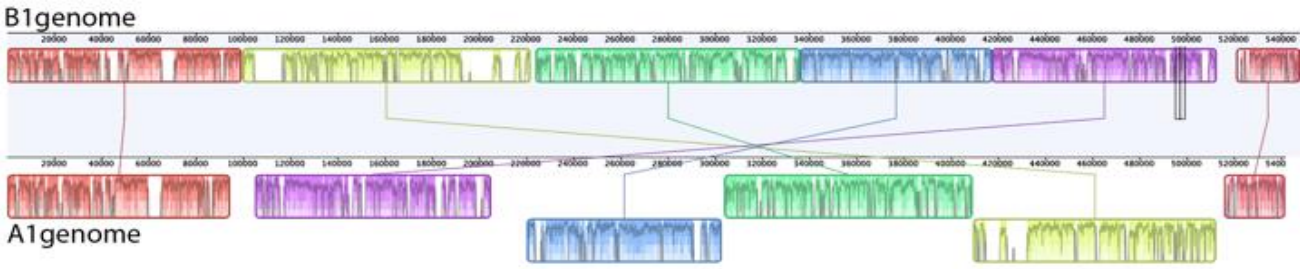

Supplementary Figure 10: Diagram illustrating the rearrangements in the B1 relative to A-1, 2 and C1 genomes. Blocks of the same color are syntenous.

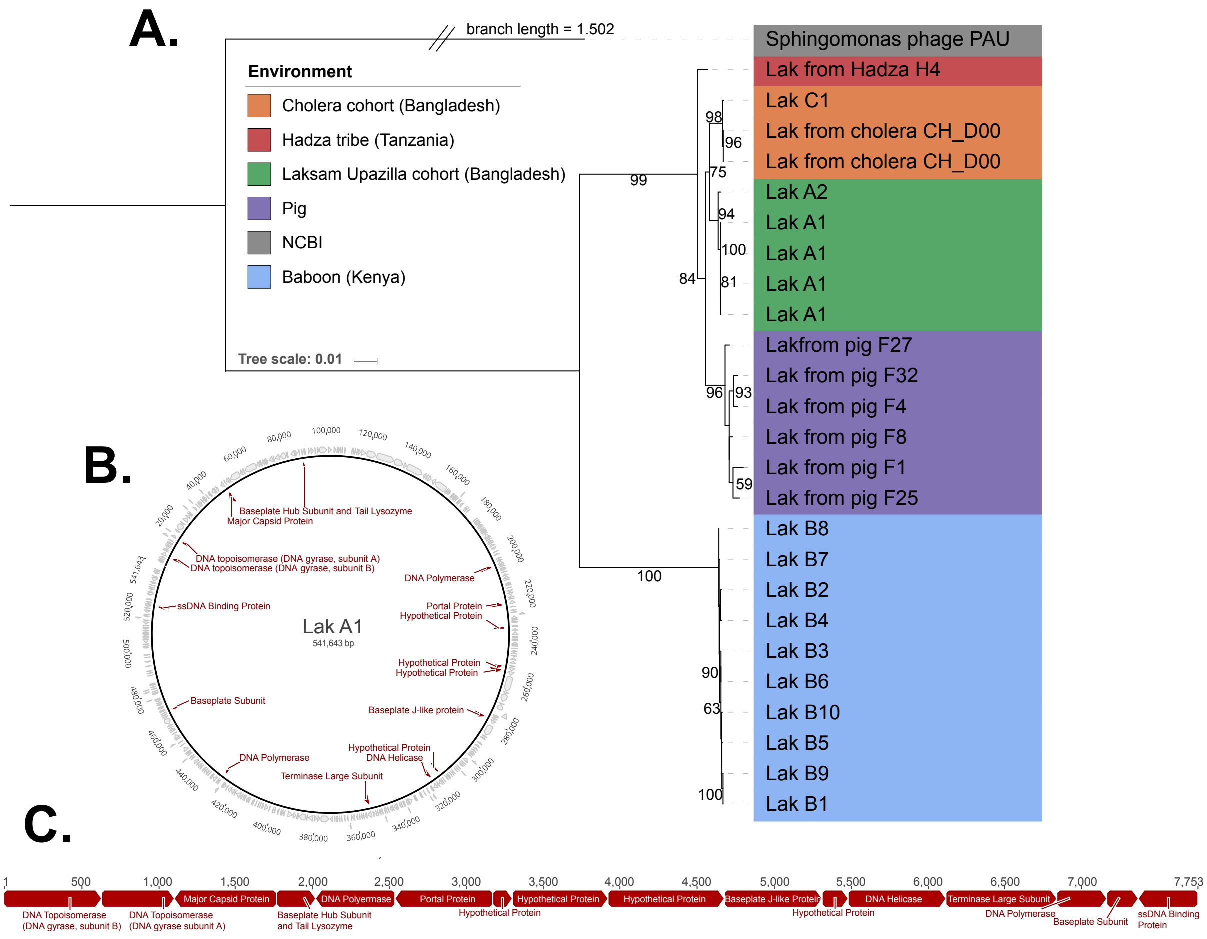

Supplementary Figure 11: Phylogenetic analysis based on a concatenation of 16 predicted proteins conserved between all available Lak genomes and bins and *Sphingomonas* phage PAU. **A.** RAxML tree generated from a concatenation of the 16 conserved proteins. Branch lengths represent the number of amino acid substitutions per site; note the PAU outgroup branch is significantly longer than the branches within the Lak clade and has been truncated. Label colorColored bars indicates the study each lak genome or bin was found in. Bootstrap values >50 shown. **B.** Location on the A1 genome of the conserved proteins used in the concatenation. **C**. Gene order, strand, and relative length of the proteins used in the concatenation (order based on A1 genome, same as in B).

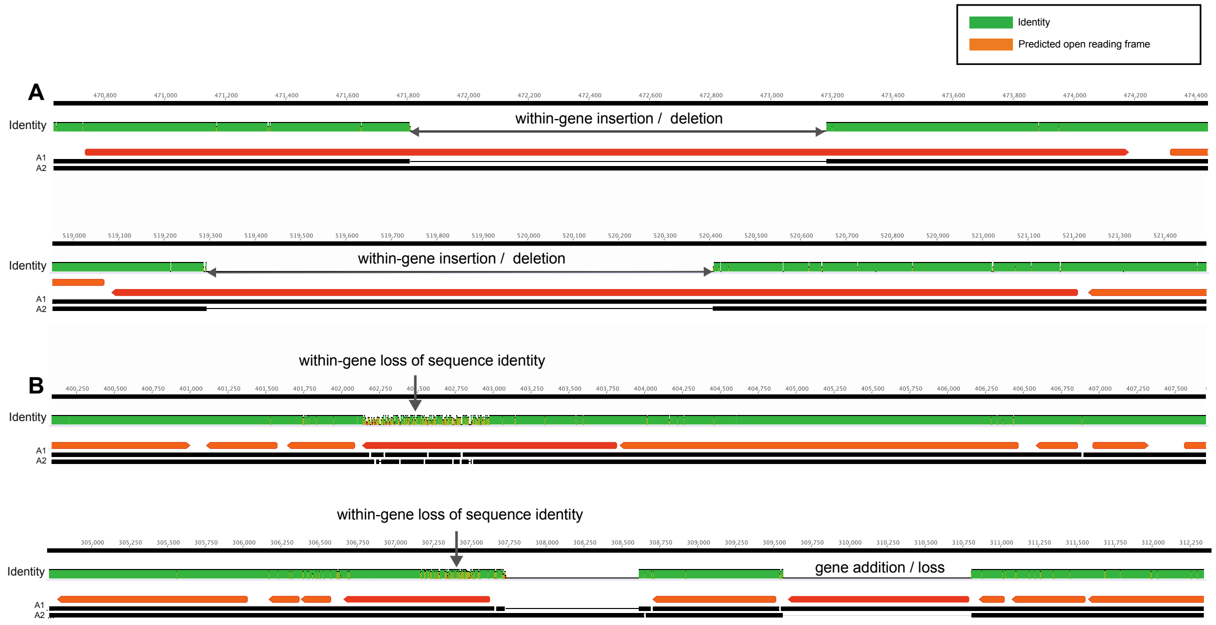

Supplementary Figure 12: Examples of phenomena that distinguish the A1 and A2 genomes. **A.** Within-gene deletions, examples of which are also apparent in **Figure 2** alignments of all Lak genomes with A1. **B.** Sudden loss of sequence identity within a gene and an example of a gene insertion / loss that distinguishes the genomes.

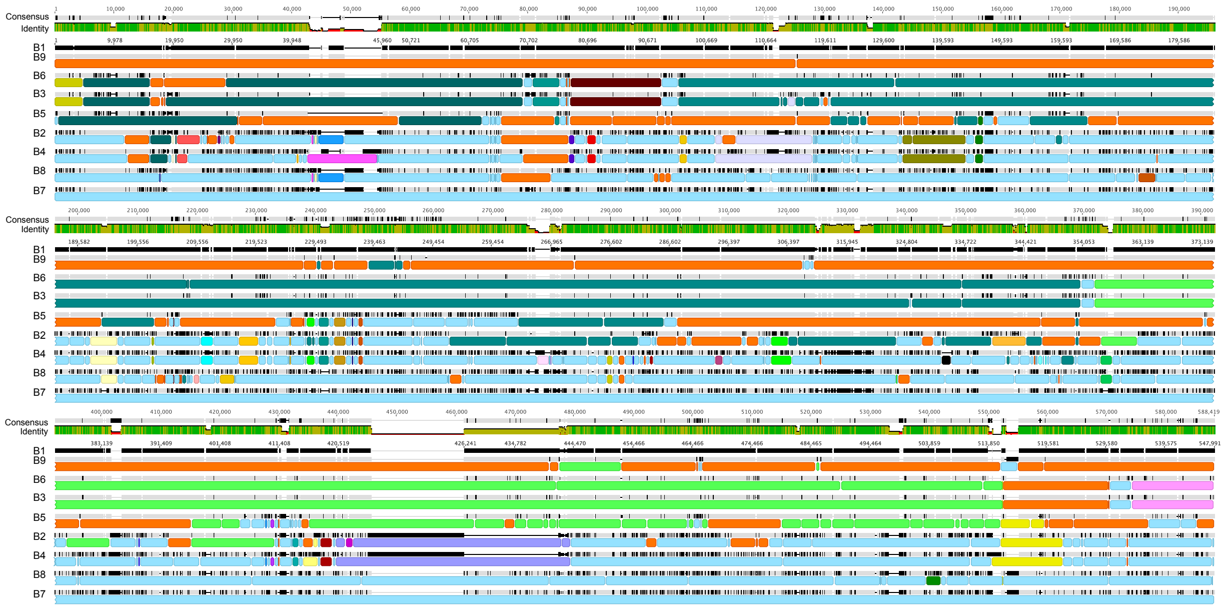

Supplementary Figure 13: Alignment of the nine complete B-Lak genomes, with B1 (top black line) set as the reference sequence. Each genome is a row and vertical black tick marks indicate polymorphic sites. The sequence of B1 was designated as type ‘orange’ and sequence B7 as type ‘blue’. Sequence blocks of the same color Colored underlines indicate sequence blocks that are identical. Small gaps within bars of the same color indicate SNPs that are not in agreement despite flanking sequence identity. Notably, the patterns indicate extensive admixture of sequence blocks, presumably due to homologous recombination, and often involve the available B-Lak genomes. For example, blocks of sequence shared with B7 (light blue underlines) occur in all B-Lak genomes. One region within the lowest panel is shown in detail in **Figure 3.**

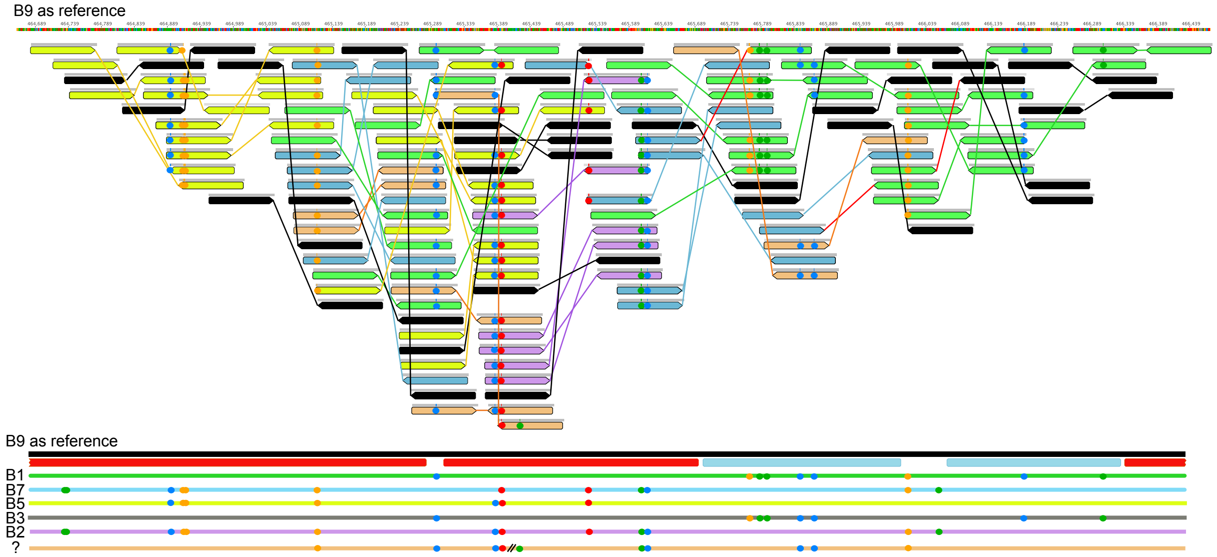

Supplementary Figure 14: Genotypic variation within the B9 population. The upper panel shows a small subset of reads (light grey bars underlined with colored bars) that mapped to a hypervariable region of the B9 genome. Example paired reads were selected for display as they carried replicated SNPs (colored circles highlight SNP positions and types). As shown by red and blue bars in the lower panel, this region overlaps with that analyzed in **Figure 3**. Each read was assigned to a possible genotype (in some cases, multiple choices were possible), indicated by the color of the reads (top panel) so as to match to the color of the genome sequences in the lower panel. Some paired read variants could be assigned to the B7 genome, extending backward from the small region where B9 and B7 are identical. Many variant reads could be assigned to B1, perhaps not surprising given the overall high similarity between the B1 and B9 genomes. Others could be assigned to B2 and/or B5. A subset of read pairs had SNP combinations that were distinct from those of known genomes, supporting the inference of recombination with as yet unreconstructed B-Lak genotypes. Despite the relative clonality of most other regions, these data indicate that the phage population includes rare genotypes that reflect homologous recombination, mostly involving sequences found in the B-Lak genome collection. Some paired reads contain combinations of SNPs not found in the B-Lak genomes (cream color). This may reflect recombination with other B-Lak genotype(s).

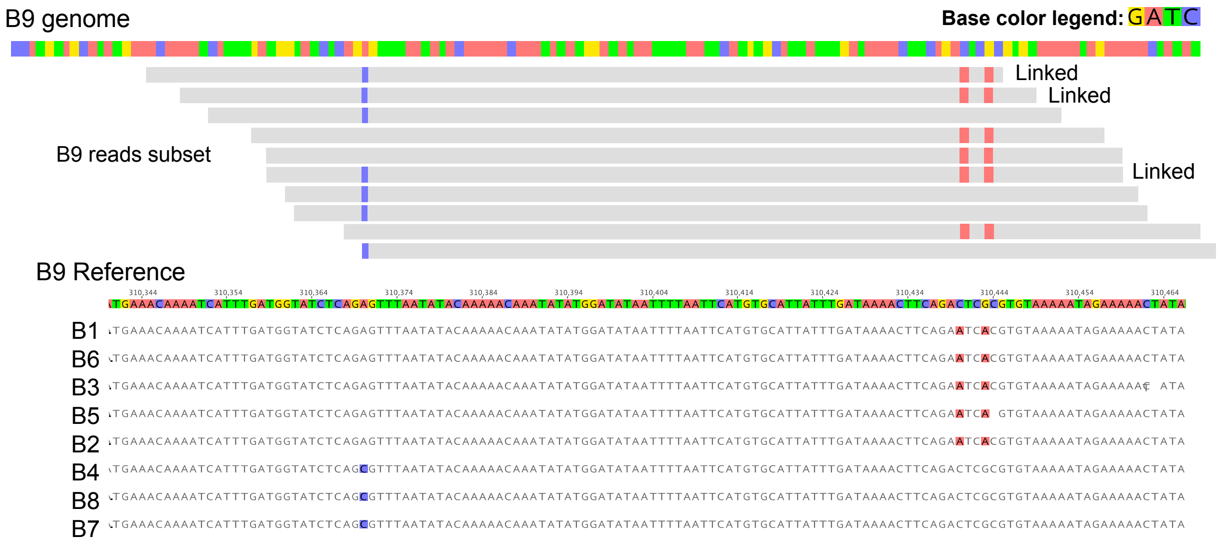

Supplementary Figure 15: Diagram showing SNP-bearing single reads that span adjacent polymorphic sites and the comparison of these sequences to the B1-B9 genome sequences at this locus. Blue vertical bars are A to C mutations, red vertical bars are C to A mutations. Note that the blue and pair of red SNPs are not linked in the B1-B9 genomes but are linked in three sequencing reads. Thus, there is no single choice for the consensus sequence at this locus. The varied SNP linkage patterns are inferred to indicate homologous recombination.

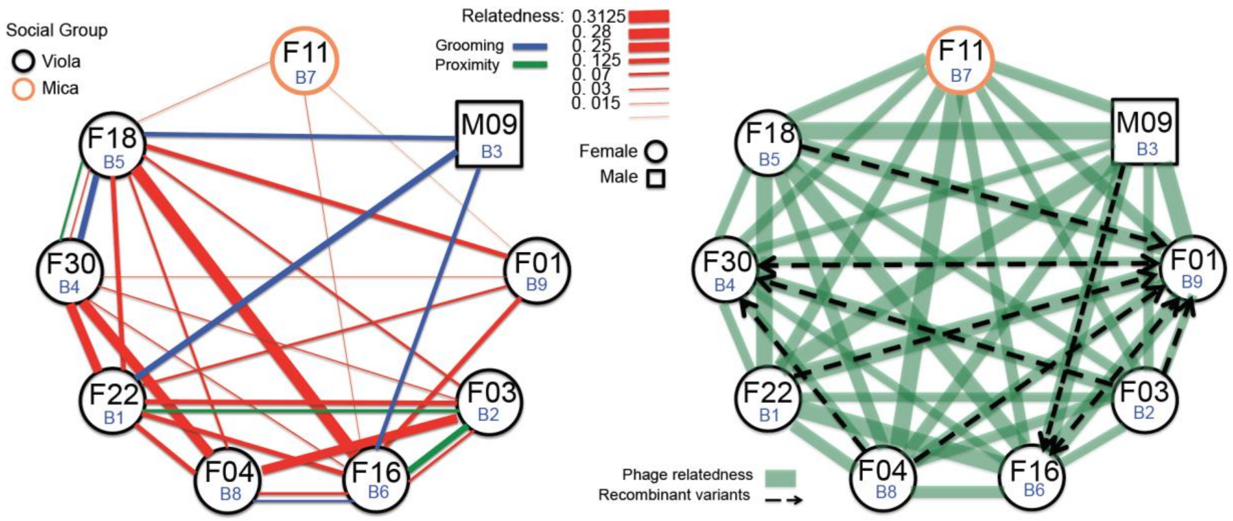

Supplementary Figure 16: Summary of the metadata for the baboons and information about the relatedness of the phage populations. Left panel shows the baboon genetic relatedness (red lines), based on a partial Mantel test for taxonomic composition as reported in (15),and social interactions (blue and green lines). All but one baboon comes from "Viola's social group". Right panel diagrams pairwise measures of phage genome sequence relatedness based on average nucleotide identity (ANI). Thicker lines indicate higher ANI. Recombinant variants refers to cases where more than about 0.1% of reads have SNPs that perfectly match sequences of another population (indicated by the arrow tip). In general, the baboon relatedness data do not predict phage relatedness or genotypic admixtures. For example, both F11 and M09 are essentially unrelated to the other baboons, as shown in the left panel, but their phage are about as strongly related to the phage of other baboons as are the phage in highly related baboons. Grooming interactions do not appear to lead to higher phage relatedness than occurs in the absence of these interactions. Similarly, proximity does not appear to be an important driver of phage relatedness (only strongest grooming and proximity links are shown but the full data are available in **Supplementary Table 5**). Baboon metadata reported by (15).

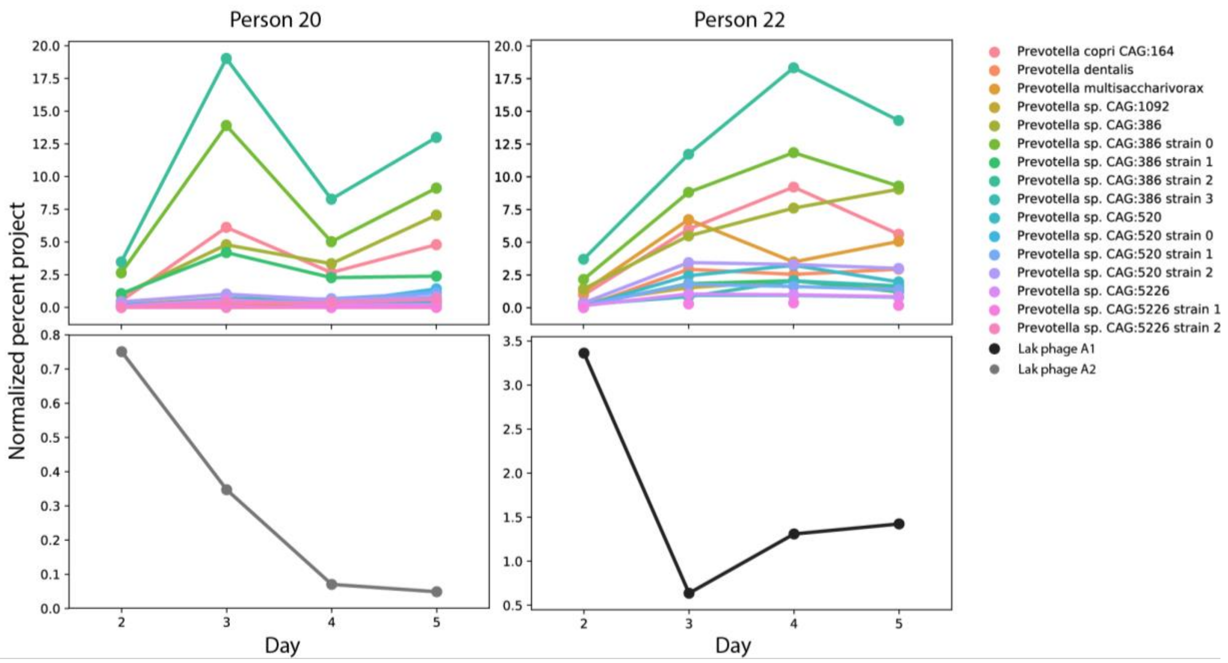

Supplementary Figure 17: Diagrams showing the changing abundances of potential *Prevotella* hosts (top panel) and Lak phage (lower panel) over the four-days sampling period for Subject 20 and Subject 22 of the cohort of Laksam Upazila, Bangladeshi adults.

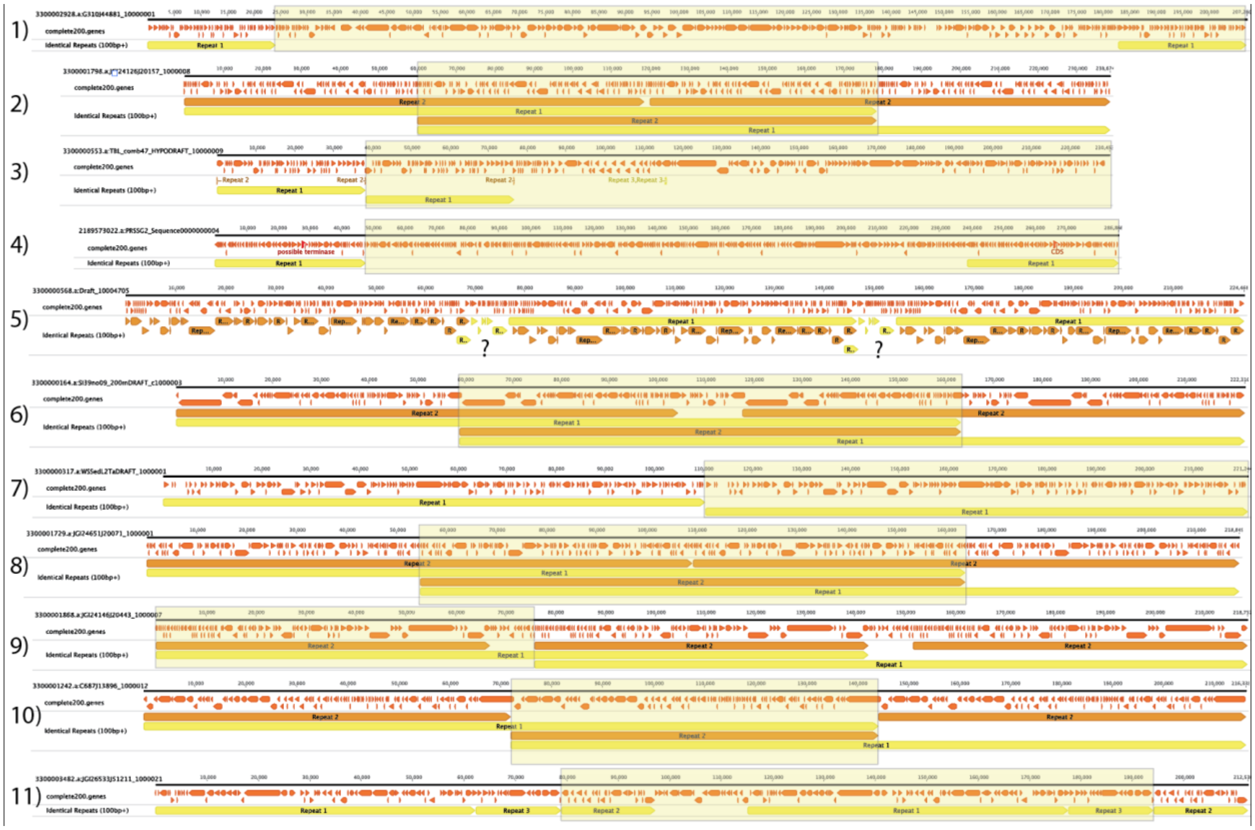

Supplementary Figure 18: Analysis of questionable assemblies of reported >200 kbp phage genomes (6). Based on large perfect repeats, we suggest that these sequences are improbable. Gold highlights indicate our suggested alternative (and much shorter, in all but one case <200 kbp) chromosome choices.

Supplementary Table 1: Sources of data used in this study.

Supplementary Table 2: Comparison of tRNAs across the Lak megaphage genome set. **A**. The tRNAs in samples organized based on overall similarity in tRNA gene content. Note that some tRNA predictions are not very reliable (e.g., Sup tRNAs and pseudo-tRNAs) due to the large divergence between phage sequences and well studied tRNAs. **B.** The tRNAs in the Lak phage genomes listed based on position in the genome. Heavy black underlines indicate rearrangements to establish syntenic blocks

Supplementary Table 3: Table of average nucleotide identity (ANI) of phage genomes shared with A1, calculated over the aligned regions shown in **Figure 3B. B.** Lak phage ANI % over the full genome alignments.

Supplementary Table 4: The fraction of reads from each sample that map perfectly to the genome recovered from that sample and mapping of sequences carried on reads that do not map perfectly to other B-Lak genomes. Also shown is the fraction of reads that cannot be mapped to any reconstructed genome. An example mapping illustrates the case where unclassifiable SNPs tile out an otherwise unknown variant sequence. For more explanation, see **Supplementary Discussion**.

Supplementary Table 5: Baboon metadata. **A.** Baboon pedigree scores, **B.** Grooming interactions, **C**. Proximity scores. Data from (15). Based on **A**. and information in **Supplementary Table 3**, genetic relatedness of baboons does not predict relatedness of phage, based on a correlation analysis.

Supplementary Table 6: Sequences and other information related to tRNA introns.

Supplementary Table 7: Genes with functional predictions. **A**. Listing of genes in the A1 Lak phage genome with a confident, moderately confident (purple functional prediction) or lower confidence (grey text) prediction. Structural proteins are listed in blue text. **B**. Genes with functional annotations from **Supplementary Table 7A** sorted by function.

Supplementary References

1. Kurtz S, et al. (2004) Versatile and open software for comparing large genomes. *Genome Biol* 5(2):R12.

2. Yuan Y, Gao M (2017) Jumbo Bacteriophages: An Overview. *Front Microbiol* 8:403.

3. Dutilh BE, et al. (2014) A highly abundant bacteriophage discovered in the unknown sequences of human faecal metagenomes. *Nat Commun* 5:4498.

4. Hayashi H, Shibata K, Sakamoto M, Tomita S, Benno Y (2007) Prevotella copri sp. nov. and Prevotella stercorea sp. nov., isolated from human faeces. *Int J Syst Evol Microbiol* 57(Pt 5):941–946.

5. Bailly-Bechet M, Vergassola M, Rocha E (2007) Causes for the intriguing presence of tRNAs in phages. *Genome Res* 17(10):1486–1495.

6. Paez-Espino D, et al. (2016) Uncovering Earth's virome. *Nature* 536(7617):425–430.

7. Yamada T, et al. (2010) A jumbo phage infecting the phytopathogen Ralstonia solanacearum defines a new lineage of the Myoviridae family. *Virology* 398(1):135–147.

8. Yoshikawa G, et al. (2018) Xanthomonas citri jumbo phage XacN1 exhibits a wide host range and high complement of tRNA genes. *Sci Rep* 8(1):4486.

9. Abbasifar R, et al. (2014) Supersize me: Cronobacter sakazakii phage GAP32. *Virology* 460-461:138–146.

10. Das U, Shuman S (2013) Mechanism of RNA 2',3'-cyclic phosphate end healing by T4 polynucleotide kinase-phosphatase. *Nucleic Acids Res* 41(1):355–365.

11. Ambrozic J, Ferme D, Grabnar M, Ravnikar M, Avgustin G (2001) The bacteriophages of ruminal prevotellas. *Folia Microbiol* 46(1):37–39.

12. Rampelli S, et al. (2015) Metagenome Sequencing of the Hadza Hunter-Gatherer Gut Microbiota. *Curr Biol* 25(13):1682–1693.

13. David LA, et al. (2015) Gut microbial succession follows acute secretory diarrhea in humans. *MBio* 6(3):e00381–15.

14. Ghosh TS, et al. (2014) Gut microbiomes of Indian children of varying nutritional status. *PLoS One* 9(4):e95547.

15. Tung J, et al. (2015) Social networks predict gut microbiome composition in wild baboons. *Elife* 4. doi:10.7554/eLife.05224.

16. Obregon-Tito AJ, et al. (2015) Subsistence strategies in traditional societies distinguish gut microbiomes. *Nat Commun* 6:6505.

17. Thomas M, et al. (2017) Metagenomic characterization of the effect of feed additives on the gut microbiome and antibiotic resistome of feedlot cattle. *Sci Rep* 7(1):12257.
