# Supplementary material for: Megaphages infect Prevotella and variants are widespread in gut microbiomes
Source: Nat Microbiol. 2019 Jan 28;4(4):693–700. doi: 10.1038/s41564-018-0338-9 (PMC6784885; doi:10.1038/s41564-018-0338-9)
Supplement: Supplementary file 2 — Reporting Summary [file 41564_2018_338_MOESM2_ESM.pdf]

nature research

Corresponding author(s): Jillian F. Banfield

Reporting Summary

Nature Research wishes to improve the reproducibility of the work that we publish. This form provides structure for consistency and transparency in reporting. For further information on Nature Research policies, see Authors & Referees and the Editorial Policy Checklist.

Statistical parameters

When statistical analyses are reported, confirm that the following items are present in the relevant location (e.g. figure legend, table legend, main text, or Methods section).

n/a	Confirmed	
☐	☒	The exact sample size (n) for each experimental group/condition, given as a discrete number and unit of measurement
☐	☒	An indication of whether measurements were taken from distinct samples or whether the same sample was measured repeatedly
☒	☐	The statistical test(s) used AND whether they are one- or two-sided<br>*Only common tests should be described solely by name; describe more complex techniques in the Methods section.*
☒	☐	A description of all covariates tested
☒	☐	A description of any assumptions or corrections, such as tests of normality and adjustment for multiple comparisons
☐	☒	A full description of the statistics including central tendency (e.g. means) or other basic estimates (e.g. regression coefficient) AND variation (e.g. standard deviation) or associated estimates of uncertainty (e.g. confidence intervals)
☒	☐	For null hypothesis testing, the test statistic (e.g. F, t, r) with confidence intervals, effect sizes, degrees of freedom and P value noted<br>*Give P values as exact values whenever suitable.*
☒	☐	For Bayesian analysis, information on the choice of priors and Markov chain Monte Carlo settings
☒	☐	For hierarchical and complex designs, identification of the appropriate level for tests and full reporting of outcomes
☒	☐	Estimates of effect sizes (e.g. Cohen's d, Pearson's r), indicating how they were calculated
☒	☐	Clearly defined error bars<br>*State explicitly what error bars represent (e.g. SD, SE, CI)*

Our web collection on statistics for biologists may be useful.

Software and code

Policy information about availability of computer code

Data collection	Geneious 9.1.8 (Licensed, paid version used in this study, free versions available)<br>BBmap (Version: Last modified May 11, 2017)<br>IDBA_UD 1.1.1<br>Bowtie2 aligner 2.3.4.1
Data analysis	Prodigal V2.6.3<br>usearch v10.0.240_i86linux64, 1057Gb RAM, 80 cores<br>tRNAscan-SE 2.0<br>MUSCLE v3.8.31<br>blastn: 2.6.0+ (command line version)<br>CRISPRDetect 2.2 (command line version)<br>Mummer 4.0.0beta<br>cu.py (available at https://github.com/oddaud/cu.py)

For manuscripts utilizing custom algorithms or software that are central to the research but not yet described in published literature, software must be made available to editors/reviewers upon request. We strongly encourage code deposition in a community repository (e.g. GitHub). See the Nature Research guidelines for submitting code & software for further information.

Data

Policy information about availability of data

All manuscripts must include a data availability statement. This statement should provide the following information, where applicable:

- Accession codes, unique identifiers, or web links for publicly available datasets
- A list of figures that have associated raw data
- A description of any restrictions on data availability

The 15 Lak phage genomes have been deposited at NCBI under BioProject PRJNA491720. The genomes can also be downloaded from https://ggkbase.berkeley.edu/project_groups/megaphage. Read archive and other accession information is provided in Table S1. Please note that it is necessary to register for a ggKbase account by providing an email address prior to accessing or downloading the data.

Field-specific reporting

Please select the best fit for your research. If you are not sure, read the appropriate sections before making your selection.

☒ Life sciences ☐ Behavioural & social sciences ☐ Ecological, evolutionary & environmental sciences

For a reference copy of the document with all sections, see nature.com/authors/policies/ReportingSummary-flat.pdf

Life sciences study design

All studies must disclose on these points even when the disclosure is negative.

Sample size	Pig cohort: N=105 samples, where each sample is the pooled feces of multiple individual pigs<br>Laksam, Bangladesh cohort: N = 10 male adults, 3-4 samples per adult<br>Cholera-impacted cohort: N = 42 male adults, 3 female adults, 2 male and 2 female children<br>Baboon cohort: 17 male adults and 31 female adults
Data exclusions	None
Replication	The A1 genome was independently reconstructed from four samples collected on consecutive days. All curated assemblies were verified in multiple read mapping steps.<br>Sample collection was not replicated except when multiple samples were collected from the same adult in the Laksam, Bangladesh cohort. Samples from the same individual were more similar to each other than other individuals, as expected.
Randomization	Randomization is not applicable because there were no experimental groups designated in this study.
Blinding	Blinding was not performed because it was not applicable to this study. This study was a survey of various populations, and was not dependent on the presence / absence of certain characteristics.

Reporting for specific materials, systems and methods

Materials & experimental systems

n/a	Involved in the study	
☒	☐	Unique biological materials
☒	☐	Antibodies
☒	☐	Eukaryotic cell lines
☒	☐	Palaeontology
☒	☐	Animals and other organisms
☐	☒	Human research participants

Methods

n/a	Involved in the study	
☒	☐	ChIP-seq
☒	☐	Flow cytometry
☒	☐	MRI-based neuroimaging

Human research participants

Policy information about studies involving human research participants

Population characteristics	The only human subjects used in this study where from the Laksam, Bangladesh cohort. All other human and animal subjects were described by previous studies that had been published at the time of writing this paper.

The Laksam, Bangladesh cohort consisted of 10 adult male subjects that were currently hospitalized. All subjects displayed signs of arsenicosis and were consuming arsenic-contaminated drinking water.

Recruitment	All patients were recruited do to their presentation of arsenicosis symptoms. Given that they were not healthy individuals and were in a diseases state, it is unknown how their disease contributed to the results of this study. However, the Lak phage reported here were found in numerous other environments including in livestock, so it is unlikely that the health of these individuals played a significant role in the presence/absence of Lak. Their health may have, however, played a role in the overall composition of their microbiome.
